# Single‐Molecule Binding Assay Using Nanopores and Dimeric NP Conjugates

**DOI:** 10.1002/adma.202103067

**Published:** 2021-07-29

**Authors:** Ren Ren, Maozhong Sun, Pratibha Goel, Shenglin Cai, Nicholas A. Kotov, Hua Kuang, Chuanlai Xu, Aleksandar P. Ivanov, Joshua B. Edel

**Affiliations:** ^1^ Department of Chemistry Molecular Science Research Hub Imperial College London White City Campus, 82 Wood Lane London W12 0BZ UK; ^2^ Key Lab of Synthetic and Biological Colloids Ministry of Education State Key Lab of Food Science and Technology International Joint Research Laboratory for Biointerface and Biodetection School of Food Science and Technology Jiangnan University Wuxi Jiangsu 214122 P. R. China; ^3^ Department of Chemical Engineering University of Michigan Ann Arbor MI 48109 USA; ^4^ Department of Materials Science and Engineering University of Michigan Ann Arbor MI 48109 USA

**Keywords:** diagnostics, molecular probes, nanopore sensing, screening, self‐assembly, single‐molecule detection

## Abstract

The ability to measure biomarkers, both specifically and selectively at the single‐molecule level in biological fluids, has the potential to transform the diagnosis, monitoring, and therapeutic intervention of diseases. The use of nanopores has been gaining prominence in this area, not only for sequencing but more recently in screening applications. The selectivity of nanopore sensing can be substantially improved with the use of labels, but substantial challenges remain, especially when trying to differentiate between bound from unbound targets. Here highly sensitive and selective molecular probes made from nanoparticles (NPs) that self‐assemble and dimerize upon binding to a biological target are designed. It is shown that both single and paired NPs can be successfully resolved and detected at the single‐molecule nanopore sensing and can be used for applications such as antigen/antibody detection and microRNA (miRNA) sequence analysis. It is expected that such technology will contribute significantly to developing highly sensitive and selective strategies for the diagnosis and screening of diseases without the need for sample processing or amplification while requiring minimal sample volume.

## Graphical abstract

A single‐molecule nanopore‐based sensing strategy is developed to screen for biomarkers. In the presence of the target biomolecules, AuNPs dimerize and are detected electrically using a nanopore. This approach is highly sensitive and selective and can potentially be used for diagnosis and screening of diseases without the need for sample processing or amplification.

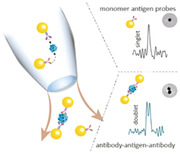

## 1 Introduction

Early‐stage screening and rapid diagnosis are essential steps in improving the effectiveness of therapeutic intervention. To this end, there has been a considerable drive to develop ultrasensitive biomarker detection strategies with single‐molecule sensitivity. With such tools, it becomes possible to screen for targets with very low abundance within complex media such as serum, urine, and cerebral spinal fluid. One approach that has been gaining attention is nanopore sensing. DNA sequencing and nucleic acid detection have been the major driving force,^[^ ^1^ ^]^ more recently, there has been progress in using nanopores for the detection and sensing of protein,^[^ ^2^ ^]^ nanoparticles,^[^ ^3^ ^]^ neurotransmitters,^[^ ^4^ ^]^ and other biomolecules.^[^ ^1^, ^5^ ^]^ The popularity is in large part due to the simplicity of the approach. Briefly, molecules are translocated one at a time through a nanometer‐sized pore using an applied electric field across an insulating membrane, that separates two electrolyte‐filled reservoirs.^[^ ^1^, ^6^, ^7^ ^]^ An ionic current is generated by the applied electric field and the passage of ions through the nanopore. Individual analytes are detected by observing changes in the transients of the ionic current. The magnitude, dwell time, and frequency of these transients reveal information such as size,^[^ ^2^, ^8^ ^]^ charge,^[^ ^9^ ^]^ shape,^[^ ^8^, ^10^ ^]^ dipole,^[^ ^11^ ^]^ concentration,^[^ ^12^ ^]^ and in the case of nucleic acids, even the sequence.^[^ ^13^ ^]^ However, the detection of proteins adds significant complexity both in terms of sensitivity and selectivity. Proteins, unlike nucleic acids, have a heterogeneous charge and typically translocate faster than can be detected, and when they do, they often exhibit a lower signal‐to‐noise. Besides, the detection of biomolecules with nanopores is generally sensitive to the volume and the surface charge of the analytes, and improvements in the method are needed to accurately distinguish between molecules with very similar size and charge composition. To date, much effort has been invested to remedy these issues, which include functionalization of the nanopore lumen with binding moieties,^[^ ^14^ ^]^ incorporation of field‐effect transistors,^[^ ^15^ ^]^ and use of high‐bandwidth instruments,^[^ ^16^ ^]^ to name a few. One of the most promising strategies is based on the use of molecular carriers, or probes, such as NPs^[^ ^17^ ^]^ and DNA.^[^ ^12^, ^18^ ^]^

These probes can be designed to bind to a target biomarker selectively (usually using a grafted aptamers or antibodies) and subsequently translocate through the nanopore for detection. When a biomarker bound to the molecular probe passes through the nanopore, a multilevel current signal is often observed, with the first level originating from the probe and the secondary level caused by the biomarker. A number of groups, including our own, have already utilized DNA‐based molecular probes to detect specific biomolecules with high sensitivity.^[^ ^12^, ^18^, ^19^ ^]^ However, it is often difficult to distinguish between the secondary signal arising due to a partially folded DNA probe and that of the target analyte. In addition, when the size of the target analyte is small, the secondary level can be masked by background noise. To address these limitations, we have designed a series of molecular probes based around the use of gold NPs, which have shown that it is possible to design dimeric NPs linked by either dsDNA^[^ ^20^ ^]^ or antigen/antibodies.^[^ ^21^ ^]^

**Figure 1:**
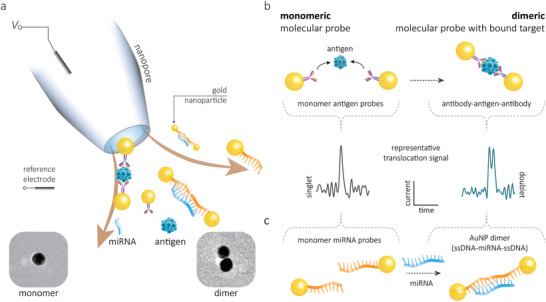
a) Schematic of the platform and a cartoon representation of monomeric and dimeric NPs translocated though the nanopore. The ionic current signal can be used to differentiate between AuNP monomers and dimers and as a result selectively determine the concentration of target analytes. b) Monomeric NPs (17–20 nm) modified with antibodies can be used to initiate self‐assembly and dimerization in the presence of an antigen. This can be quantified by measuring the number of doublets versus singlets in the translocation signal. Nanopore diameter: 26 ± 4 nm. c) A similar strategy can be used for the selective detection of miRNA. Two populations of NPs can be modified to contain half of the complementary miRNA sequence. In the presence of miRNA the monomeric NPs will self‐assemble and dimerize. By quantifying the dimerized fraction it becomes possible to determine the analyte concentration.

**Figure 2:**
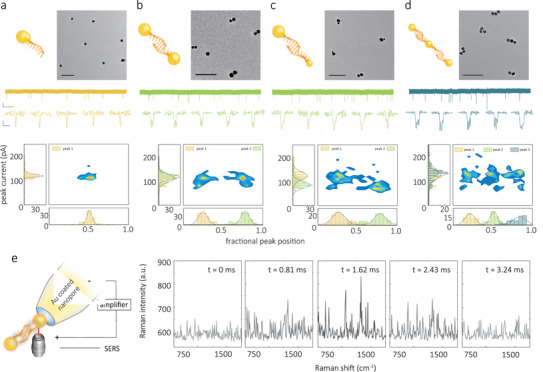
a–d) TEM images (scale bar: 100 nm), and current–time traces for AuNP monomers (a), AuNPs symmetrical dimers (b), AuNPs asymmetrical dimers (c), and AuNPs trimers (d). The scale bar for the current–time trace is 50 pA (vertical) and 5 s (horizontal). Typical individual translocation events are also shown and have a scale bar of 50 pA and 20 µs. All the translocation experiments were performed in 50 × 10^−3^ m KCl, 10 × 10^−3^ m Tris‐EDTA, and at a voltage of −600 mV. Surface density plots of peak current versus fractional peak position show that the translocation signal can differentiate between monomers, dimers, and even trimers. e) Single‐molecule SERS was also used to confirm NP dimerization along with translocation through the nanopipette. The NPs were coated with 4‐aminothiophenol and the nanopipette was coated in gold to maximize SERS enhancement. The spectra were recorded using a dwell time of 810 µs, and translocation experiments were performed at −800 mV. An example of a sequence of SERS spectra for a single 4 ms translocation event is shown.

Our strategy relies on differentiating between monomeric and dimeric NP probes that are highly sensitive to the presence of proteins or miRNA, **Figure** **1**. The target analyte triggers the self‐assembly and dimerization of the monomeric NPs. Distinguishing monomers and dimers is inherently simple as only a single peak in the translocation signal would be observed for a monomeric NP. In contrast, a dimeric NP produces a doublet and hence confirms the presence of the target biomarker. A unique advantage of this strategy is that it becomes possible to sense relatively small molecules, not easily detected with solid‐state nanopores, as the signal originates from the NP rather than the target analyte. We validated this strategy with two classes of molecular probes: 1) antigen bound NPs for detection of procalcitonin (PCT) essential for the diagnosis of sepsis^[^ ^22^ ^]^ (Figure 1b); 2) nucleic acid bound NPs for the detection of short miRNA sequences which are upregulated in patients with active prostate cancer^[^ ^23^ ^]^ (Figure 1c). By measuring the ratio between the detected monomers and dimers, we show that it is possible to determine the concentration of the biomarker with high sensitivity and selectivity at the single‐molecule level.

Importantly both strategies are fully adaptable to studying alternative targets by functionalizing the NP with almost any available antibody or nucleic acid sequence.

## 2 Result and Discussion

### 2.1 Qualification of AuNP Monomers, Dimers, and Trimers

To validate the screening ability of the AuNP conjugates, we first confirmed that the experimental set‐up had sufficient resolution to differentiate between monomeric and dimeric NPs. Initially, the spacing between NPs was controlled by using double‐stranded DNA spacers, **Figure** **2**. AuNP symmetrical dimers (Figure 2b) were fabricated by self‐assembly of two 17 ± 3 nm AuNP monomers, each consisting, on average, of 1.5 single thiolated DNA strand (10 base linker and 15 bases complementary sequence). Upon hybridization, the NPs were separated by a 35 base dsDNA spacer. To further assess the spatial resolution, asymmetric dimers consisting of 10 and 20 nm AuNP monomers were also used, Figure 2c. Finally, trimers were also synthesized and assembled by controlling the NP monomer ratio, Figure 2d. Detailed synthesis protocols and schematics of the molecular probe designs used can be found in Note S1 and Figure S1, Supporting Information respectively. The geometry and size of the nanostructures were confirmed by transmission electron microscopy (TEM), Figure 2a–d. All NP conjugates were dispersed after synthesis in a 50 × 10^−3^ m KCl, 10 × 10^−3^ m Tris‐EDTA buffer. The stability was confirmed by monitoring the UV–vis spectra after 24 h and prior to nanopore experiments. The ionic strength of the solutions was optimized to be close to physiological conditions while minimizing NP aggregation and maximizing the signal‐to‐noise of the translocation signal.

Nanopore experiments were performed with nanopipettes fabricated by laser‐assisted pulling using single‐barreled quartz capillaries.^[^ ^24^ ^]^ The nanopores, ≈25 nm in diameter, were pulled to be on average slightly larger than the diameter of the NPs, as measured by scanning electron microscopy (SEM), Figure S2, Supporting Information. These dimensions closely matched the diameters (26 ± 4 nm) estimated from nanopore conductance measurements,^[^ ^25^ ^]^ 15.3 ± 2.4 nS in 100 × 10^−3^ m KCl (*n* = 18), Figure S3, Supporting Information. From SEM imaging, the taper angle of the nanopipette tip was measured to be 16.9 ± 1.1° over the first 100 nm (*n* = 18), Figure S4, Supporting Information, which allowed us to estimate the effective sensing length to be between 25–50 nm, calculated based on a 75–80% resistance drop at the nanopore (Note S2, and Figures S5,S6, Supporting Information). The analyte was filled inside the nanopipette, where an AgCl patch electrode was placed. A ground/reference AgCl electrode was placed in the bath, outside the pipette. It was possible to transport the AuNPs from inside (*cis*) to outside (*trans*) of the nanopipette by applying a negative voltage. Chronoamperometric traces (*I*–*t*) were recorded at clamped voltage using a high bandwidth amplifier (Chimera Instruments, VC100) with a 1 MHz sampling rate and a 100 kHz low‐pass digital filter.

At low salt concentration, small, charged conical nanopores exhibit ion‐perm selectivity and significant ion concentration polarization.^[^ ^26^ ^]^ This phenomenon leads to a transient current increase when species with high surface charge density such as DNA or AuNP are translocated through the nanopore. In our case, current transients (net negative current increase at negative voltage) corresponding to the translocation of single NPs were recorded based on their dwell time and current amplitude increase. A comparison of the translocation characteristics for the different conjugates, including representative traces, individual translocation events, and scatter plots and histograms of dwell time and peak current, is shown in Figure 2. The nanopore translocation of the simplest constructs, AuNP monomers, resulted in relatively quick events with a mean dwell time of 8 ± 1 µs at a voltage of −600 mV, and a current distribution with a single peak (singlet), Figure 2a and Figure S7, Supporting Information.

The distribution of the dwell times was normalized, with 0 being defined as the translocation onset and 1 being defined as the end of the translocation.^[^ ^12b^ ^]^ This allowed us to take into account the difference in dwell times from translocation to translocation and compare the fractional position of the current peak maxima or the translocation time between two monomers in a doublet. Symmetric and asymmetric dimers with a 35 base DNA spacer showed fractional position plots with an apparentdoublet distribution, Figure 2b,c, and comprised dwell times just over twice in duration compared to that of the monomer (Figure S7, Supporting Information). The doublets observed in the current amplitude were consistent with the translocation of the dimers in a linear conformation, that is, the first peak appears as a net current amplitude increase due to the translocation of the AuNP. A decrease in current follows as a result of the transport of the linker through the nanopore. Finally, a second peak appears as the second AuNP in the dimer translocates through the nanopore. Notably, one could distinguish between the doublet peaks with high temporal resolution even though the spacing was only 28–51 nm in length, depending on the probe design. Further studies on the voltage dependence and comparison between AuNP symmetrical dimers with different linker lengths (35 bases and 115 bases) are shown in Figures S8,S9, Supporting Information, respectively.

For asymmetric dimers, Figure 2c, the size of the individual NPs was reflected in the shape and the current amplitude for each peak in the doublet. As an example, peak currents of 155.5 ± 21.6 and 81.8 ± 13.5 pA corresponded to the translocation of the 20 and 10 nm AuNP in the dimers. Interestingly, 92 ± 4% of all translocation events of asymmetric dimers showed a preferential orientation with the larger NP being transported first, which was attributed to the larger NPs carrying higher surface charge. We also investigated the possibility of translocating and detecting NP trimers, Figure 2d, although these fall beyond the scope of this study. Trimers could be detected and resolved, as observed with a typical triplet signature in the ionic current and three distinct populations in the fractional peak position. Trimers, on average, took 33 ± 5 µs to translocate through the nanopore, which is 3.94‐fold and 1.57‐fold longer than the respective dwell times for monomers and dimers. This is in good agreement due to a change in spatial length for which the trimer is 4.23‐fold and 1.61‐fold longer than the monomer and dimer, respectively. Measurements containing a mixture of monomer, dimer, and trimers were also performed, and clear single, double, and triple events could be observed (Figure S10, Supporting Information).

To further characterize the translocations of these NPs, single‐particle surface‐enhanced Raman scattering (SERS)^[^ ^27^ ^]^ was also performed on a modified nanopore coated with 10 nm‐thick gold, Figures S11,S12, Note S3, Supporting Information. Due to the coupling and proximity between the dimers and the surface of the nanopore, a significant enhancement in the Raman signal could be obtained.^[^ ^28^ ^]^ To achieve single‐particle SERS, somewhat larger 35 nm, AuNP symmetrical dimers were used due to the higher scattering cross‐section. The AuNPs were functionalized with 4‐aminothiophenol (ATP) dye, Figure 2e. The dimer was further stabilized with poly(ethylene glycol) (PEG) to ensure the particles do not aggregate at the 100 × 10^−3^ m salt concentrations required to perform the translocations. A typical SERS spectrum of the NPs in bulk solution is shown and consists of expected peaks at 1138, 1387, and 1571 cm^–1^, Figure S13, Supporting Information. This is comparable to the data obtained for single‐particle SERS, seen from the transients, Figure 2e. In this example, the dwell times recorded optically were 3.24 ± 0.81 ms, which is longer than the corresponding electrical events (0.79 ± 0.29 ms), Figure S14, Supporting Information. The longer optical dwell times were attributed to the diffraction‐limited laser spot size (≈1 µm) and the optical detection volume being significantly larger than the nanopore sensing region. As a negative control, Raman spectra that show no Raman signal, were also acquired when a reverse voltage was applied, Figure S15, Supporting Information. We envisage that this method can also be used to perform molecular assays and complement the electrical detection shown in this manuscript and simultaneous electro‐optical sensing, as previously published by our group.^[^ ^12^, ^29^ ^]^ In the future, combined nanopore sensing with SERS single‐molecule detection can likely provide an additional modality that is particularly useful in the context of multiplexed detection using large nanopore arrays.

### 2.2 Molecular Probes for Single‐Molecule Detection of miRNA

miRNAs are a class of short noncoding RNAs that function in RNA silencing and post‐transcriptional gene regulation. Besides their participation in regulating normal physiological activities, specific miRNA types could act as oncogenes, tumor suppressors, or metastasis regulators, and have emerged as promising biomarkers for cancer. Conventional methods include Northern blotting, in situ hybridization, RT‐qPCR, or microarrays. However, these methods require sample preparation or processing. Also, each technique has specific limitations such as low throughput and low sensitivity (for northern blotting), semi‐quantitative (for in situ hybridization), time‐consuming, specific reaction conditions (for RT‐qPCR), high cost, and relatively low accuracy (for microarrays). Recent advances in nanopore technology offer the promise of addressing some of these drawbacks for detecting miRNA with high sensitivity and selectivity.^[^ ^30^ ^]^ However, the signal of these short fragments (typically 18–23 bases) is hard to detect directly with solid‐state nanopores due to the high‐speed translocation and low signal‐to‐noise ratio, Figure S16, Supporting Information. Here, we use AuNP dimer self‐assembly to amplify this translocation signal, leading to very efficient miRNA detection at the single‐molecule level.

In this study, we use AuNP molecular probes for the detection of miR‐141‐3p. miR‐141 is commonly dysregulated in malignant tumors such as those associated with prostate cancer and plays essential roles in tumor development and progression and has emerged as a potential biomarker of prostate cancer.^[^ ^31^ ^]^ Prostate cancer is the second most common cancer in men worldwide; however, disease outcome is difficult to predict in large part due to the lack of efficient diagnostic strategies. As such, miR‐141‐3p has the potential to become a useful biomarker.

**Figure 3:**
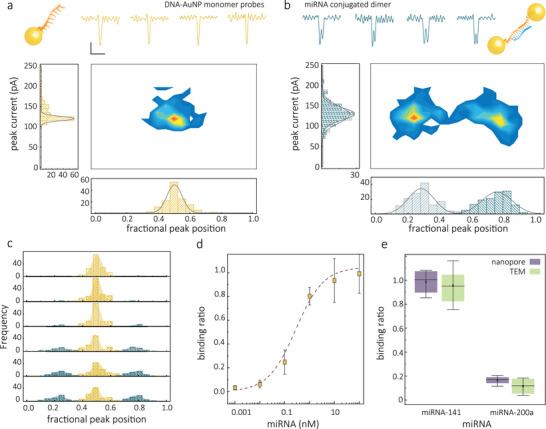
Nanopore sensing of mRNA‐141. a) AuNP monomer miR‐141‐3p molecular probes. Representative individual events are shown (scale bar: vertical 50 pA, horizontal 20 µs) along with associated statistics. b) Conjugated dimers with miRNA‐141 linked between 2 NP monomers. All the nanopore experiments were performed in 50 × 10^−3^ m KCl, 10 × 10^−3^ m Tris‐EDTA, and at a voltage of −600 mV. c) The normalized peak position of the monomer probe and conjugated dimer with different concentrations of mRNA‐141 (from top to bottom, 0 × 10^−9^ m, 10 × 10^−12^ m, 100 × 10^−12^ m, 1 × 10^−9^ m, 10 × 10^−9^ m, and 100 × 10^−9^ m). d) The binding curve of 2 × 10^−9^ m AuNP monomer miRNA‐141 probes incubating with the target miRNA ranging from 0 to 100 × 10^−9^ m. e) The comparison of the detection of miR‐141‐3p and miR‐200a‐3p using AuNP monomer miR‐141‐3p probes. The error bars represent the standard deviation of three independent experimental repeats.

The molecular probes consisted of two populations of ssDNA functionalized to AuNP monomers. Each of them was modified by an 11 base recognition sequence, which can hybridize with half of the 22‐base‐long miR‐141‐3p, Figure S17, Supporting Information. With the addition of the target, the monomer probes self‐assemble to form dimers and produce doublet signatures, **Figure** **3**a–c. A binding assay was performed within the miRNA concentration range of 1 × 10^−12^ m to 100 × 10^−9^ m. The number of dimers, hence doublets, increases with concentration Figure 3d,e. Dimer formation is validated and compared with TEM, Figure S18, Supporting Information, providing visual evidence of dimer formation due to the presence of miR‐141‐3p. Typically, the concentration of miR‐141‐3p is between × 10^−15^ and × 10^−12^ m in unprocessed prostate cancer patient samples and between × 10^−12^ and × 10^−9^ m in extracted miRNA samples.

The specificity of the molecular probes was verified by the detection of miR‐141‐3p and miR‐200a‐3p using AuNP monomer miR‐141‐3p probes. Both miR‐141 and miR‐200a are in the miR‐200 family and share seed sequences differing in only two nucleotides, Figure S19, Supporting Information. Detection of miR‐141‐3p gives a significant binding ratio, calculated from the ratio of detected dimers, whereas the control experiment, detecting the miR‐200a‐3p, leads to a low value of the binding ratio, Figure 3e. These results were in excellent agreement with dimer formation and binding ratios obtained from TEM measurements (see Figure 3e, and Figure S20, Supporting Information). The specificity can be tuned by the dimerization mechanism. For example, in our case, for miR‐141‐3p, the monomer probes can be linked to the dimer because the ssDNA is fully matching the target. In contrast, for the miR‐200a‐3p, the two mismatch points happened on the same ssDNA of one monomer probe, leading to a very low binding affinity, which causes unsuccessful dimerization. This result shows that the AuNP monomer probe can detect the target with high specificity.

### 2.3 Molecular Probes for Single‐Molecule Detection of PCT

**Figure 4:**
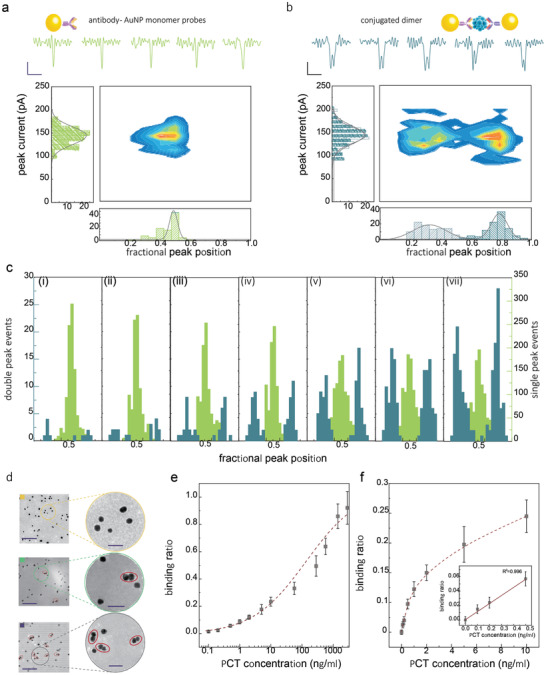
Nanopore sensing of PCT. a) AuNP monomeric molecular probes consisting of a single antibody. Individual translocation events are shown (Scale bar: vertical 50 pA, horizontal 20 µs) along with associated statistics. b) Conjugated dimers linked by an antibody‐antigen‐antibody sandwich. All the translocation experiments were performed in 50 × 10^−3^ m KCl, 10 × 10^−3^ m Tris‐EDTA, and at a voltage of −600 mV. c) Normalized peak position of the monomer probe and conjugated dimer at varying PCT concentration: i) 0.1 ng mL^−1^, ii) 0.2 ng mL^−1^, iii) 0.5 ng mL^−1^, iv) 1.0 ng mL^−1^, v) 2.0 ng mL^−1^, vi) 5.0 ng mL^−1^, vii) 10.0 ng mL^−1^. d) TEM images are used to confirm successful binding (scale bar: 200 nm, enlarged, 50 nm), from top to bottom, 0 ng mL^−1^, 1.0 ng mL^−1^, 2.9 µg mL^−1^ PCT, respectively. e) The binding curve of 2 × 10^−9^ m AuNP monomer PCT probes incubating with PCT ranging from 0 to 2.9 µg mL^−1^. f) Same binding curve as in (e), highlighting the clinically relevant range. A linear increase in the binding ratio is observed at low concentrations, as shown in the inset. The error bars represent the standard deviation of three independent experimental repeats.

As mentioned earlier, the detection of relatively small molecules (some small proteins, peptides, etc.) selectively using nanopores can be challenging. Molecular probes can be used in part to tackle this issue. However, the smaller the target, the progressively harder it gets to detect the difference in signal originating from the molecular probe and peak associated with the analyte, Figure S22, Supporting Information. Building on our previous work,^[^ ^21^ ^]^ a universal strategy for sensing small antigen molecules has been developed. Here we used a mixture of AuNPs with half the population being modified with an antibody (mAb1) and another half being modified with another antibody (mAb2). In the presence of the antigen, the AuNPs self‐assemble and dimerize upon binding to the two antibodies. The presence and concentration or absence of the antigen can be confirmed by comparing the number of dimers (doublet peaks) versus monomers (singlet peaks), **Figure** **4**a,b.

In this example, we synthesize AuNP modified with antibody probes for the detection of PCT(*M*_W_ 14.5 kDa; pI of 6.5), which is a peptide precursor of the hormone calcitonin. Measuring the PCT level in patients has become an important indicator of identifying bacterial infections and guiding antibiotic therapy.^[^ ^32^ ^]^ It is, therefore, essential to detect PCT at trace levels below 10 ng mL^−1^. A comparison between the translocation of 2 × 10^−9^ m AuNP monomers (50% are functionalized by PCT mAb1, and 50% are functionalized by mAb2) and the assembled dimer complex after adding 0.29 µg mL^−1^ (≈20 × 10^−9^ m) PCT is shown in Figure 4a,b. Without the presence of PCT, only singlets were observed during the translocation of 2 × 10^−9^ m AuNP monomers with 50 × 10^−3^ m KCl, pH 8. Upon addition of 0.29 µg mL^−1^ PCT, approximately 15% of the singlet peaks become doublets. The double peak signature results from the translocating of two AuNPs linked by the mAb‐PCT‐sAb sandwich linker (PI of mAb1, mAb2 is 6.6–7.2). As a negative control, no doublets were observed when PCT was replaced by other antigens such as insulin, Figure S24, Supporting Information.

To validate that this sandwich immunoassay can be used at clinically relevant concentrations,^[^ ^22b^ ^]^ a binding assay was performed at varying PCT concentrations. In this case, the concentration of the molecular probes was kept at 2 × 10^−9^ m, while the concentration of PCT was varied from 0 to 10.0 ng mL^−1^. As expected, the percentage of singlets decreased, whereas the proportion of doublets increased with the addition of PCT, Figure 4c. The results were further confirmed by TEM (Figure 4d; Figures S25,S26, Supporting Information), providing visual evidence of binding and NP assembly. The full binding curve with PCT ranging from 0 to 2.9 µg mL^−1^ is shown in Figure 4e.

Notably, at high analyte concentrations, higher‐order NP aggregates were not observed, which was attributed to the effective 1:1 ratio between antibody and NP. It is important to note that all binding curves saturated at approximately 33% and was consistent with different measurement techniques. This is due to an equal probability of the solution containing two forms of the monomeric NPs bound with PCT, and dimeric NPs linked with PCT, Figure S26, Supporting Information. All binding ratios were therefore normalized to the maximum value of 33%.

In bacterial infections, the concentration of PCT in plasma increases from 0.15 to more than 10 ng mL^−1^ with increasing severity of the disease. At the same time, PCT has also been used to guide antibiotic therapy; for example, if PCT levels are less than 0.1 ng mL^−1^, antibiotic therapy is strongly discouraged; if PCT levels are greater than 1 ng mL^−1^, antibiotic therapy is strongly encouraged.^[^ ^22b^ ^]^ To validate that our probes have sufficient sensitivity, the limit of detection (LOD) was calculated from the linear range in Figure 4f and was determined to be 0.12 ng mL^−1^. To further validate the method can be used in a complex solution, we also performed the experiments in 1% BSA solution. The result indicates that dimers can still be discriminated at high interferent protein concentration, Figure S27, Supporting Information. It should be noted that the target antigen should have two different epitopes so that the nanoparticle dimers can be formed.

## 3 Conclusion

We have demonstrated that it is possible to design molecular probes for the selective sensing of individual targets using nanopores. The assay is based on the dimerization of individual monomeric NPs in the presence of a biomarker. We show that this strategy can be used for both protein and miRNA detection at the single‐molecule level. We validated our strategy for two applications: 1) detection of procalcitonin, a biomarker of sepsis at ultralow concentrations, and 2) highly specific detection of miR‐141‐3p, which is a potential indicator of prostate cancer. Moreover, by correlating monomer to dimer ratio, single‐molecule binding and the concentration of the analytes can be accessed.

This approach is independent of biomarker size and, in principle, can be equally efficient for the detection of both small and larger biomarkers alike. The excellent selectivity and affinity of antibody‐antigen reaction and ssDNA‐RNA base‐pairing allow the application of these strategies to diagnostics for detecting biomarkers in trace amounts. Compared to other methods, the integration of NP assemblies with nanopore technologies leads to a much‐needed combination of high‐performance detection and exceptionally small sample volumes (several microliters and below). Importantly the method is fully adaptable to studying alternative targets by functionalizing the NP with any desired antibody or nucleic acid sequence.

## 4 Experimental Section

### Fabrication of the Nanopipettes

Single‐barrel quartz capillaries (o.d., 1.0 mm, i.d., 0.7 mm, Intracell) were plasma cleaned (Harrick Plasma) and pulled using a laser‐based pipette puller (P‐2000, Sutter Instruments). A two‐line program was used (heat 800, filament 4, velocity 30, delay 170, and pull 80; heat 825, filament 3, velocity 20, delay 145, and pull 130) to produce nanopipettes with a diameter of approximately 25 nm. It should be noted that the above pulling parameters are instrument‐specific, and variations will exist from puller to puller.

### Assembly of AuNP‐Based Nanostructures

Traditional methods of bioconjugation were used to modify the NPs.^[^ ^33^ ^]^ Details of the synthesis of AuNP monomers, AuNP symmetrical dimers (35 bases or 115 bases linker), AuNP asymmetrical dimers, AuNP trimers, and 4‐ATP modified dimers are shown in Note S2, Supporting Information.

### Preparation of Monomer PCT Probes

The antibody for PCT (mAb) was prepared in the authors’ lab. Initially, the antibody was obtained by a eukaryotic expression system and then immunized the mice. This was followed by screening and measuring the selectivity and affinity between the antigen and antibody. AuNPs were then functionalized with mAb and bound via electrostatic interactions. 2 mL AuNPs (2 × 10^−9^ m, 20 ± 3 nm) were centrifuged for 10 min at 8000 rpm and then resuspended in 200 µL of 10 × 10^−3^ m phosphate buffer (PB) solutions, which was adjusted to pH 9 with 0.1 m K_2_CO_3_. Next, 100 µL of the AuNPs were conjugated with anti‐PCT mAb1 (10 µL, 100 µg mL^−1^), and the other 100 µL AuNPs were modified with anti‐PCT mAb2 (10 µL, 100 µg mL^−1^), respectively. The AuNPs were blocked using a solution of BSA (10 µL, 500 µg mL^−1^). Finally, the functionalized AuNPs were centrifuged for 10 min at 7500 rpm at 4 °C. After centrifugation, the supernatant was discarded and the AuNPs were resuspended in 0.02 m Tris‐HCl, 0.1% Tween‐20, 0.1% PEG, 1% PVP, 5% sucrose, 4% trehalose, 2% sorbitol, 1% mannitol. 0.04% NaN3, and 0.2% BSA, 1 mL.

### Translocation Experiments

The buffer used in the translocation experiments consisted of 50 × 10^−3^ m KCl and 10 × 10^−3^ m Tris‐EDTA (pH = 8) unless reported otherwise. For the binding assays, 1 × 10^−9^ m molecular probes were used and incubated with the target analytes at a different concentration for at least 2 h. Approximately 10 µL of the electrolyte was filled inside the nanopipettes via a Microfil needle (MF34G, World Precision Instruments, UK). Freshly made Ag/AgCl electrodes were then inserted into the nanopipette and the bath. All ion current recording was performed using a high bandwidth amplifier VC100 (Chimera Instruments). The recorded data were resampled to 1 MHz and filtered at 100 kHz. Analysis of all translocations events was performed using custom‐written Matlab code, The Nanopore App. A workflow of the analysis procedure is shown in Section S7, Figure S28, Supporting Information.

## Conflict of Interest

The authors declare no conflict of interest.

## Author Contributions

R.R. and M.Z.S. contributed equally to this work. J.B.E., A.P.I., N.K., and C.L.X. designed and supervised the research. R.R. and M.Z.S. performed experiments. The manuscript was written through the contribution of all authors. All authors have given approval to the final version of the manuscript.

## Supporting information

Supporting Information

## Data Availability

Research data are not shared.

## References

[1] a) B. N. Miles , A. P. Ivanov , K. A. Wilson , F. Dogan , D. Japrung , J. B. Edel , Chem. Soc. Rev. 2013, 42, 15; 22990878 10.1039/c2cs35286a

[2] a) C. Plesa , S. W. Kowalczyk , R. Zinsmeester , A. Y. Grosberg , Y. Rabin , C. Dekker , Nano Lett. 2013, 13, 658; 23343345 10.1021/nl3042678 PMC4151282

[3] a) W. J. Lan , D. A. Holden , B. Zhang , H. S. White , Anal. Chem. 2011, 83, 3840; 21495727 10.1021/ac200312n

[4] D. S. Koktysh , X. R. Liang , B. G. Yun , I. Pastoriza‐Santos , R. L. Matts , M. Giersig , C. Serra‐Rodriguez , L. M. Liz‐Marzan , N. A. Kotov , Adv. Funct. Mater. 2002, 12, 255.

[5] a) H. Cai , Y. Wang , Y. Yu , M. V. Mirkin , S. Bhakta , G. W. Bishop , A. A. Joshi , J. F. Rusling , Anal. Chem. 2015, 87, 6403; 26040997 10.1021/acs.analchem.5b01468 PMC4598329

[6] a) Y. L. Ying , Y. T. Long , J. Am. Chem. Soc. 2019, 141, 15720; 31509414 10.1021/jacs.8b11970

[7] S. M. Lu , Y. Y. Peng , Y. L. Ying , Y. T. Long , Anal. Chem. 2020, 92, 5621. 32182049 10.1021/acs.analchem.0c00931

[8] X. Wang , M. D. Wilkinson , X. Lin , R. Ren , K. R. Willison , A. P. Ivanov , J. Baum , J. B. Edel , Chem. Sci. 2020, 11, 970. 10.1039/c9sc05710b PMC8146688 34084351

[9] a) H. J. Kim , U. J. Choi , H. Kim , K. Lee , K. B. Park , H. M. Kim , D. K. Kwak , S. W. Chi , J. S. Lee , K. B. Kim , Nanoscale 2019, 11, 444; 30398270 10.1039/c8nr06229c

[10] N. Varongchayakul , J. X. Song , A. Meller , M. W. Grinstaff , Chem. Soc. Rev. 2018, 47, 8512. 30328860 10.1039/c8cs00106e PMC6309966

[11] J. Houghtaling , C. F. Ying , O. M. Eggenberger , A. Fennouri , S. Nandivada , M. Acharjee , J. L. Li , A. R. Hall , M. Mayer , ACS Nano 2019, 13, 5231. 30995394 10.1021/acsnano.8b09555

[12] a) S. L. Cai , J. Y. Y. Sze , A. P. Ivanov , J. B. Edel , Nat. Commun. 2019, 10, 1797; 30996223 10.1038/s41467-019-09476-4 PMC6470146

[13] B. M. Venkatesan , R. Bashir , Nat. Nanotechnol. 2011, 6, 615. 21926981 10.1038/nnano.2011.129

[14] a) B. Luan , G. Stolovitzky , G. Martyna , Nanoscale 2012, 4, 1068; 22081018 10.1039/c1nr11201e PMC3543692

[15] a) L. Xue , P. Cadinu , B. Paulose Nadappuram , M. Kang , Y. Ma , Y. Korchev , A. P. Ivanov , J. B. Edel , ACS Appl. Mater. Interfaces 2018, 10, 38621; 30360085 10.1021/acsami.8b13721 PMC6243394

[16] a) J. Larkin , R. Y. Henley , M. Muthukumar , J. K. Rosenstein , M. Wanunu , Biophys. J. 2014, 106, 696; 24507610 10.1016/j.bpj.2013.12.025 PMC3944622

[17] a) X. Y. Lin , A. P. Ivanov , J. B. Edel , Chem. Sci. 2017, 8, 3905; 28626560 10.1039/c7sc00415j PMC5465561

[18] a) N. A. W. Bell , U. F. Keyser , J. Am. Chem. Soc. 2015, 137, 2035; 25621373 10.1021/ja512521w PMC4353036

[19] a) A. Singer , M. Wanunu , W. Morrison , H. Kuhn , M. Frank‐Kamenetskii , A. Meller , Nano Lett. 2010, 10, 738; 20088590 10.1021/nl100058y PMC2834191

[20] W. Chen , A. Bian , A. Agarwal , L. Q. Liu , H. B. Shen , L. B. Wang , C. L. Xu , N. A. Kotov , Nano Lett. 2009, 9, 2153. 19320495 10.1021/nl900726s

[21] X. L. Wu , L. G. Xu , L. Q. Liu , W. Ma , H. H. Yin , H. Kuang , L. B. Wang , C. L. Xu , N. A. Kotov , J. Am. Chem. Soc. 2013, 135, 18629. 24246036 10.1021/ja4095445

[22] a) G. A. Wanner , W. Keel , U. Steckholzer , W. Beier , R. Stocker , W. Ertel , Crit. Care Med. 2000, 28, 950; 10809265 10.1097/00003246-200004000-00007

[23] a) J. C. Brase , M. Johannes , T. Schlomm , M. Falth , A. Haese , T. Steuber , T. Beissbarth , R. Kuner , H. Sultmann , Int. J. Cancer 2011, 128, 608; 20473869 10.1002/ijc.25376

[24] a) L. M. Ying , S. S. White , A. Bruckbauer , L. Meadows , Y. E. Korchev , D. Klenerman , Biophys. J. 2004, 86, 1018; 14747337 10.1016/S0006-3495(04)74177-6 PMC1303895

[25] D. Perry , D. Momotenko , R. A. Lazenby , M. Kang , P. R. Unwin , Anal. Chem. 2016, 88, 5523. 27108872 10.1021/acs.analchem.6b01095

[26] K. K. Chen , N. A. W. Bell , J. L. Kong , Y. Tian , U. F. Keyser , Biophys. J. 2017, 112, 674. 28256227 10.1016/j.bpj.2016.12.033 PMC5340120

[27] a) M. P. Cecchini , J. Hong , C. Lim , J. Choo , T. Albrecht , A. J. Demello , J. B. Edel , Anal. Chem. 2011, 83, 3076; 21413700 10.1021/ac103329b

[28] a) J. M. Nam , J. W. Oh , H. Lee , Y. D. Suh , Acc. Chem. Res. 2016, 49, 2746; 27993009 10.1021/acs.accounts.6b00409

[29] W. H. Pitchford , H. J. Kim , A. P. Ivanov , H. M. Kim , J. S. Yu , R. J. Leatherbarrow , T. Albrecht , K. B. Kim , J. B. Edel , ACS Nano 2015, 9, 1740. 25635821 10.1021/nn506572r

[30] V. P. Dave , T. A. Ngo , A.‐K. Pernestig , D. Tilevik , K. Kant , T. Nguyen , A. Wolff , D. D. Bang , Lab. Invest. 2019, 99, 452. 30542067 10.1038/s41374-018-0143-3

[31] P. S. Mitchell , R. K. Parkin , E. M. Kroh , B. R. Fritz , S. K. Wyman , E. L. Pogosova‐Agadjanyan , A. Peterson , J. Noteboom , K. C. O'Briant , A. Allen , D. W. Lin , N. Urban , C. W. Drescher , B. S. Knudsen , D. L. Stirewalt , R. Gentleman , R. L. Vessella , P. S. Nelson , D. B. Martin , M. Tewari , Proc. Natl. Acad. Sci. USA 2008, 105, 10513. 18663219 10.1073/pnas.0804549105 PMC2492472

[32] M. Assicot , D. Gendrel , H. Carsin , J. Raymond , J. Guilbaud , C. Bohuon , Lancet 1993, 341, 515. 8094770 10.1016/0140-6736(93)90277-N PMC7141580

[33] S. P. Wang , N. Mamedova , N. A. Kotov , W. Chen , J. Studer , Nano Lett. 2002, 2, 817.

