# Supplementary material for: Single‐Molecule Binding Assay Using Nanopores and Dimeric NP Conjugates
Source: Adv Mater. 2021 Jul 29;33(38):2103067. doi: 10.1002/adma.202103067 (PMC11469134; doi:10.1002/adma.202103067)
Supplement: Supplementary file 1 — Supporting Information [file ADMA-33-2103067-s001.pdf]

© 2021 Wiley-VCH GmbH

ADVANCED MATERIALS

Supporting Information

for *Adv. Mater.,* DOI: 10.1002/adma.202103067

Single-Molecule Binding Assay Using Nanopores and Dimeric NP Conjugates

Ren Ren, Maozhong Sun, Pratibha Goel, Shenglin Cai, Nicholas A. Kotov, Hua Kuang, Chuanlai Xu, Aleksandar P. Ivanov,* and Joshua B. Edel**

Supporting Information

Single-Molecule Binding Assay Using Nanopores and Dimeric NP Conjugates

Ren Ren[1‡], Maozhong Sun[2‡], Pratibha Goel[1], Shenglin Cai[1], Nicholas A. Kotov[3,4], Hua Kuang[2], Chuanlai Xu[2], Aleksandar P. Ivanov[1*], Joshua B. Edel[1*]*

Table of contents

Section S1. Preparation of AuNP based nanostructures

Section S2. Nanopore characterization

Section S3. Quantification of AuNP monomers, dimers, and trimers

Section S4. Single-particle SERS measurement

Section S5. Molecular probes for single-molecule detection of miR-141-3p

Section S6. Molecular probes for single-molecule detection of PCT

Section S7. Data analysis

Section S1. Preparation of AuNP based nanostructures

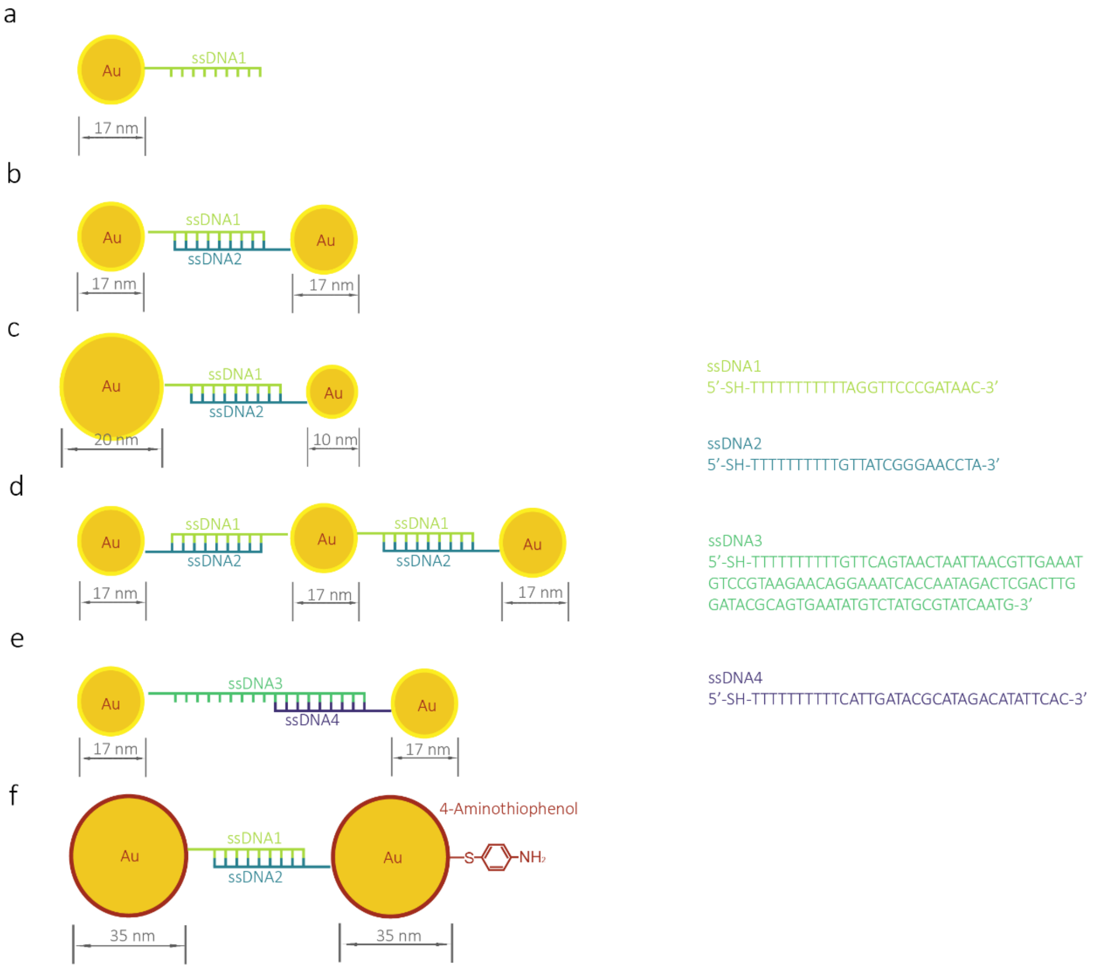

Figure S1. Schematic representation of the AuNP based probes used. (**a**) AuNP monomer consisting of a single 17 nm AuNP modified with an ssDNA. (**b**) symmetrical dimer composed of two 17 nm AuNPs linked by a DNA bridge (35 bases long). (**c**) asymmetrical dimer consisting of a 20 nm AuNP and a 10 nm AuNP linked by a DNA bridge (35 bases long). (**d**) AuNP trimer. Three AuNPs are linked by two DNA bridges (35 bases long). (**e**) Symmetrical dimer with a longer linker. Two 17 nm AuNPs are linked by a DNA bridge (115 bases long). (**f**) AuNP symmetrical dimers with Raman reporter. Two 4-aminothiophenol modified 35 nm AuNPs are linked by a DNA bridge (35 bases long). Fabrication procedures for all AuNP based probes used are shown are available in **Supplementary Notes 1**.

Supplementary Note 1. Synthesis and assembly of AuNP based nanostructures

AuNP monomer

The synthesized AuNPs (17 nm) were concentrated 10-fold and resuspended in 0.5×TBE buffer to a final concentration of 10 nM. The prepared AuNPs were then modified with the ssDNA1 (10 µM, dissolved in TE buffer) in a DNA: AuNP molar ratio of 5:1. NaCl solution (5 M) was

added to the mixture to a final NaCl concentration of 50 mM and left at 25 °C for 12 h. Finally, the mixture was centrifuged (three times) at 7000 rpm for 10 min to remove the excess DNA and resuspended in TBE buffer. The schematic of AuNP monomer is shown in **Figure S1a**. The effective binding ratio between DNA and the NP was determined to be 1.5:1.

AuNP symmetrical dimer (35 bases linker)

The process of AuNPs (17 nm) modification with the ssDNA2 sequence was the same as for DNA1. 100 μL of AuNP–DNA1 and 100 μL of AuNP–DNA2 were mixed in TBE buffer containing 50 mM NaCl. The mixture was hybridized for 12 h at room temperature under gentle stirring. The mixture was centrifuged at 5000 rpm for 10 min to remove the single nanoparticles and collect the AuNP symmetrical dimers with a short linker. The schematic of AuNP symmetrical dimer (35 bases linker) is shown in **Figure S1b**.

AuNP asymmetrical dimer

AuNPs were concentrated 10-fold and resuspended in 0.5×TBE buffer to a final concentration of 10 nM. The prepared 20 nm AuNPs were then modified with the ssDNA1 (10 μM, dissolved in TE buffer, mixture1), and 10 nm AuNPs were modified with the ssDNA2 (10 μM, dissolved in TE buffer, mixture2), in a DNA: AuNP molar ratio of 5:1. NaCl solution (5 M) was added to the mixture to a final NaCl concentration of 50 mM, and left at 25 °C for 12 h. Finally, mixture1 was centrifuged (three times) at 7000 rpm for 10 min, and mixture2 was centrifuged (three times) at 8500 rpm for 10 min to remove the excess DNA and resuspended in TBE buffer. 100 μL of 20 nm AuNP–DNA1 and 100 μL of 10 nm AuNP–DNA2 were mixed in TBE buffer containing 50 mM NaCl. The mixture was hybridized for 12 h at room temperature with gentle stirring. The mixture was centrifuged at 5000 rpm for 10 min to remove the single nanoparticles and collect the AuNP asymmetrical dimers. The schematic of the AuNP asymmetrical dimer is shown in **Figure S1c**.

AuNP trimer

100 μL of 17 nm AuNP–DNA1 and 200 μL of 17 nm AuNP–DNA2 were mixed in TBE buffer containing 50 mM NaCl. The mixture was hybridized for 12 h at room temperature with gentle stirring. The mixture was centrifuged at 4000 rpm for 10 min to remove the single nanoparticles and collect the AuNPs trimers. The schematic of AuNP trimer is shown in **Figure S1d**.

AuNP symmetrical dimer (115 bases linker)

The 17 nm AuNPs were concentrated 10-fold and resuspended in 0.5×TBE buffer to a final concentration of 10 nM. The prepared AuNPs were then modified with the ssDNA3 (10 μM, dissolved in TE buffer) in a DNA: AuNP molar ratio of 5:1. NaCl solution (5 M) was added to the mixture to a final NaCl concentration of 50 mM and left at 25 °C for 12 h. Finally, the mixture was centrifuged (three times) at 7000 rpm for 10 min to remove the excess DNA and resuspended in TBE buffer. The process of AuNPs (17 nm) modification with the ssDNA4 sequence was the same as for DNA3. 100 μL of AuNP–DNA3 and 100 μL of AuNP–DNA4 were mixed together in a TBE buffer containing 50 mM NaCl. The mixture was hybridized for 12 h at room temperature with gentle stirring. The mixture was centrifuged at 5000 rpm for 10 min to remove the single nanoparticles and collect the AuNP symmetrical dimers with the longer linker. The schematic of AuNP symmetrical dimer (115 bases linker) is shown in **Figure S1e**.

4-ATP labeled AuNP symmetrical dimers (SERS sample)

The 35 nm AuNPs were concentrated 10-fold and resuspended in 0.5×TBE buffer to a final concentration of 10 nM. The prepared AuNPs were then modified with the ssDNA1 (10 μM, dissolved in TE buffer) in a DNA: AuNP molar ratio of 5:1. NaCl solution (5 M) was added to the mixture to a final NaCl concentration of 50 mM and left at 25 °C for 12 h. Finally, the

mixture was centrifuged (three times) at 5000 rpm for 10 min to remove the excess DNA and resuspended in TBE buffer.

4-Aminothiophenol was added to the 35 nm AuNPs, with the final concentration of 10 μM. After six h, the AuNPs were centrifuged at 5000 rpm for 10 min to remove the unconnected 4-Aminothiophenol. Then, the above AuNPs were modified with ssDNA2. Finally, 100 μL of 35 nm AuNP–DNA1 and 100 μL of 35 nm AuNP–DNA2 were mixed in TBE buffer containing 50 mM NaCl. The mixture was hybridized for 12 h at room temperature with gentle stirring. The mixture was centrifuged at 3000 rpm for 10 min to remove the single nanoparticles and collect the 4-ATP labeled AuNP symmetrical dimers. To increase the stability of the large AuNPs in high salt concentration, the SERS samples are further modified by PEG-SH. The schematic of 4-ATP labeled AuNP symmetrical dimers is shown in **Figure S1f**.

Counting the numbers of DNA on AuNP

The DNA amount on AuNP was measured through a fluorescence-based method: To determine the number of DNA loaded on each AuNP, the concentration of nanoparticles was measured by UV-visible spectroscopy measurements.[1] Then the fluorescent-labeled DNA was added, and the concentration of DNA in each sample was measured by fluorescent spectrum. After that, the DNA was chemically displaced from the nanoparticle surface using dithiothreitol (DTT), and the fluorescence in solution after centrifugation.

Section S2. Nanopore characterization

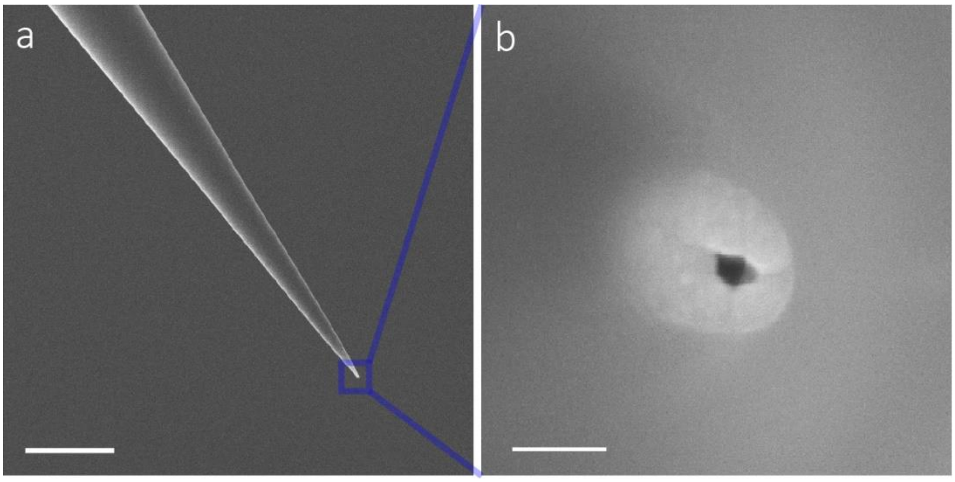

Figure S2. SEM images of a representative nanopipette. (**a**) side view (scale bar, 10 μm) and (**b**) cross-section (scale bar, 100 nm) of a nanopipette. From the images, the conical shape of the nanopipette and the nanopore located on the tip can be observed.

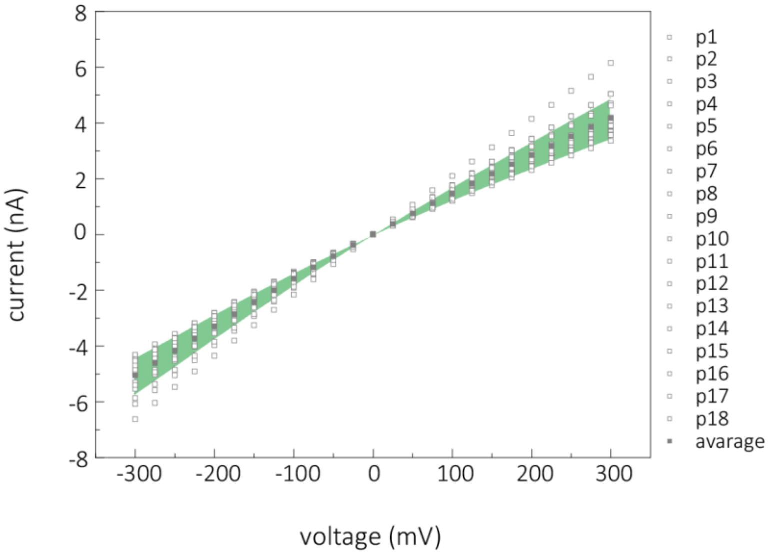

Figure S3. I-V characterization of the nanopores. The quartz nanopipettes were pulled from quartz capillaries by a laser-based pipette puller. The measurements of the I-V characterization were performed in 100 mM KCl, 10 mM Tris-EDTA, pH 8. The conductance of quartz nanopores was 15.3 ± 2.4 nS (n = 18), as calculated from the ohmic region (± 100 mV) of the I-V plots.

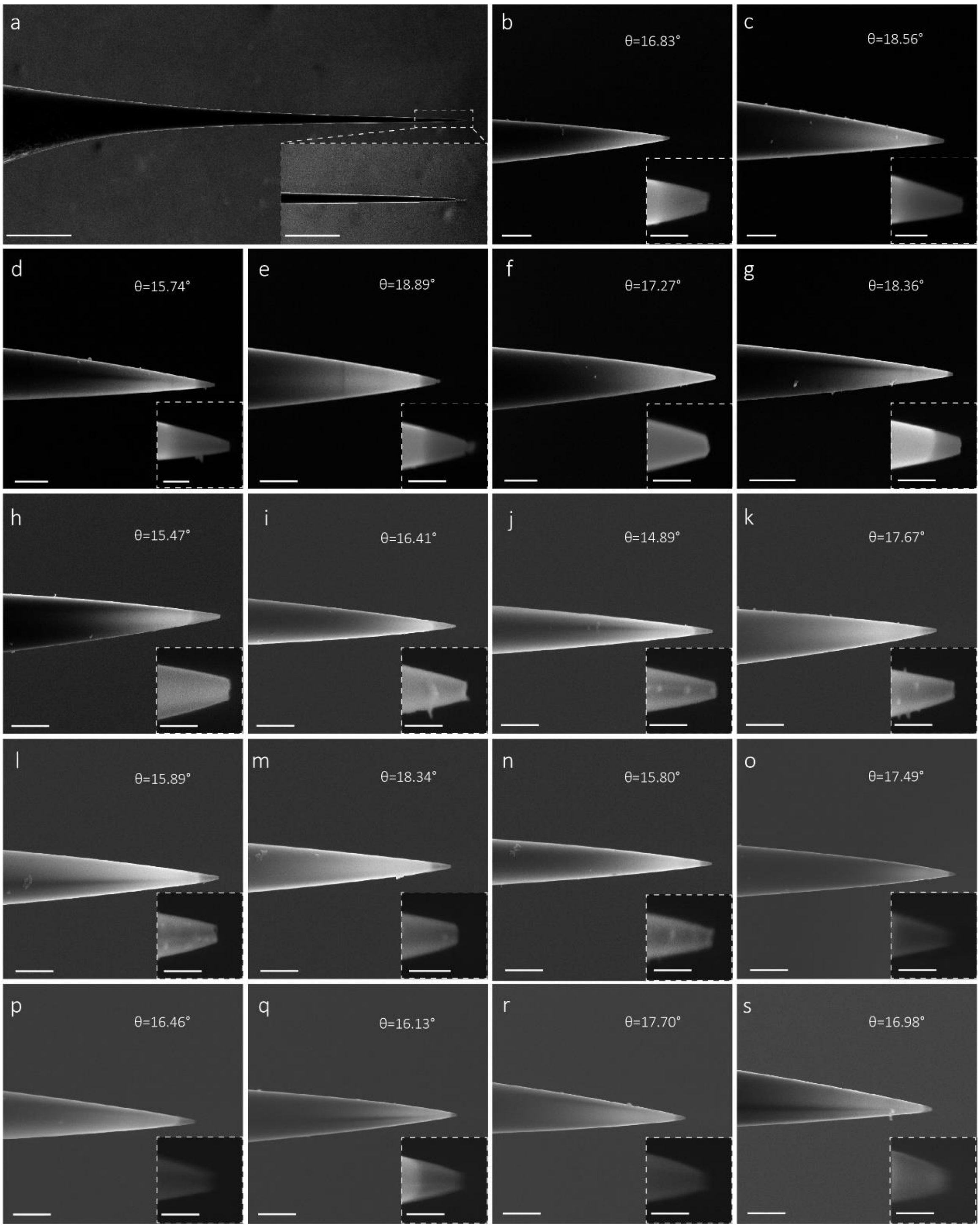

Figure S4. SEM of the nanopore and tip angle measurement. (**a**) SEM image showing the tip length and geometry (scale bar 100 µm, inset 20 µm) (**b-s**) The side view of the nanopore and the semi-angle of the first 100 nm of the nanopipette (scale bar 1 µm, inset 40 nm). The effective length of the nanopore was calculated using the parameters shown in **Supplementary Note 2** and **Figure S5-6**.

Supplementary Note 2. The calculation of the nanopipette effective sensing length

The effective sensing length of a nanopipette is the length scale over which the segment of the electrolyte-filled pore dominates the majority (for our calculations 75-80%) of the nanopore ionic resistance. The voltage drop is most significant in this segment. For a cylindrical nanopore (e.g., cylindrical solid-state nanopores in planar membranes), the pore length equals the effective length of the nanopore as the voltage drops along the pore length. However, in the conical nanopore, such as the ones located on the tip of a nanopipette, a nonlinear drop of the electric field and resistance occurs due to the increasing pore diameter D along with the nanopipette length.[2] To detect the effective sensing length, the resistance distribution along the pore axis can be estimated (**Figure S4**). The resistance Rx at different position x, ranging from 0 to L, along the length of the nanopore can be calculated. D_0 represents the diameter at the nanopore opening, and the D_L represents the diameter at a length L from the nanopore opening. D_X can be expressed as the following function: [3]

$$\mathrm{D}_x = \frac{x(D_L - D_0) + LD_0}{L} \qquad (1)$$

The resistance Rx can be calculated as:

$$\mathrm{R}_x = \frac{4L\rho}{\pi D_0 D_x} \qquad (2)$$

where ρ is the specific resistance of the electrolyte, which fills the pore. The R_{tot} represents the total resistance. Therefore, combining the equation (1) and (2), the R_X/R_{tot} versus x were plotted as **Figure S5**.

It should be noted that the model used is as a first approximation. The actual nanopipette had curved walls close to the nanopore opening, and the change of the nanopore diameter along the nanopore length was nonlinear. It can be seen from the SEM images shown in Figure S4, and the nanopore diameter decreases more closer to the nanopore opening. Schematic approximating this geometry is presented in **Figure 5Sb**.

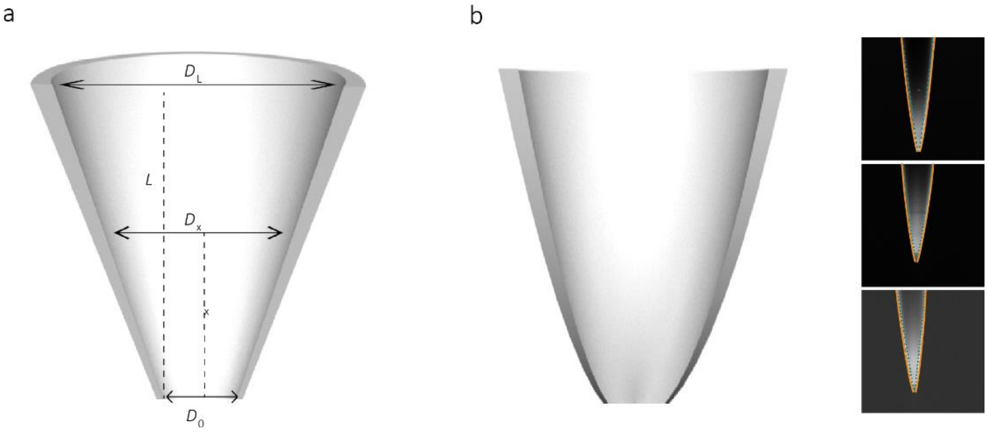

Figure S5. Schematic representation of a nanopipette. (**a**) the model of the conical nanopore for effective length calculation. (**b**) nanopore shape model. The inset shows the shape of the nanopore from SEM image. The green dash line shows the conical shape, where the solid orange line shows the contour of the nanopipette tip.

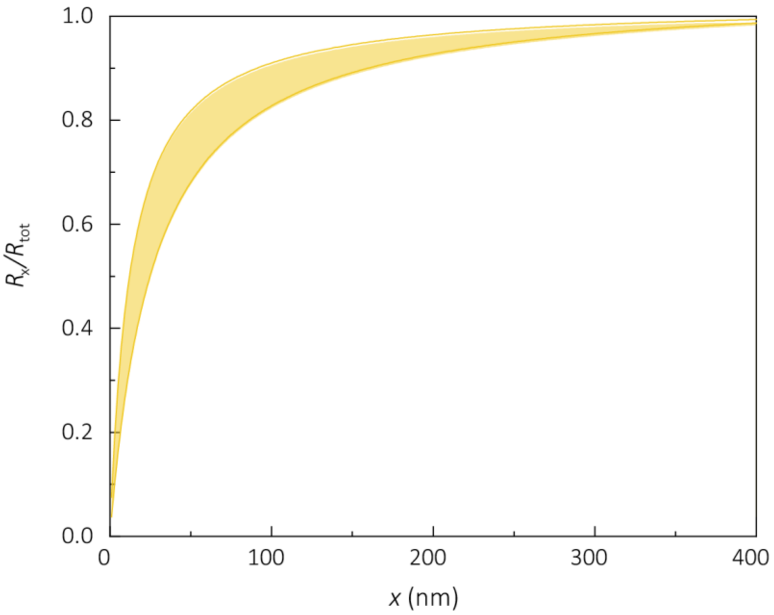

Figure S6. Calculation of the nanopipette effective sensing length. The plot shows R_X/R_{tot} at a nanopore length x for 18 conical nanopores. The effective sensing length was calculated at 75%-80%.[3b, 4]

Section S3. Quantification of AuNP monomers, dimers, and trimers

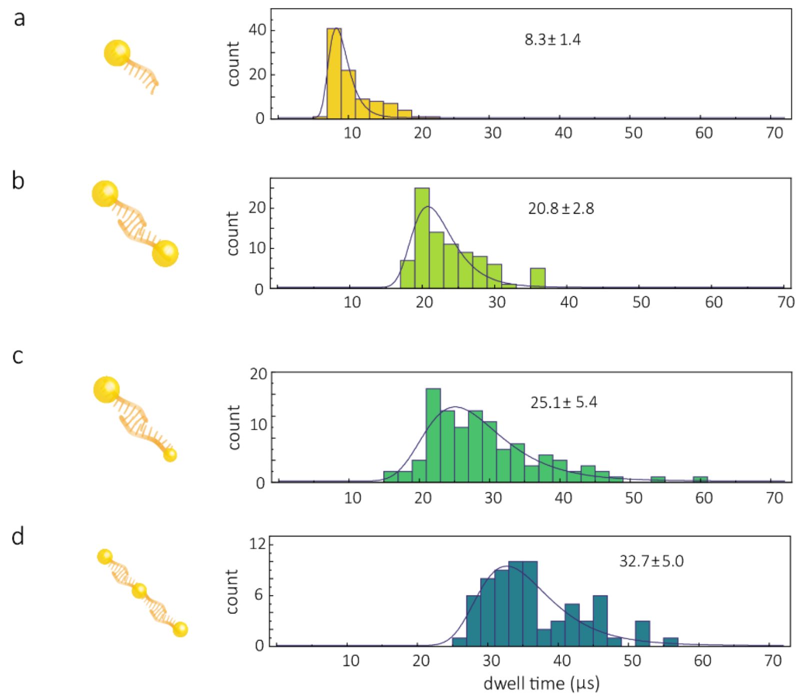

Figure S7. Dwell time for the translocation of AuNP based nanostructures. The translocation dwell time of (**a**) AuNP monomer (**b**) AuNP symmetrical dimer (**c**) AuNP asymmetrical dimer (**d**) AuNPs Trimer.

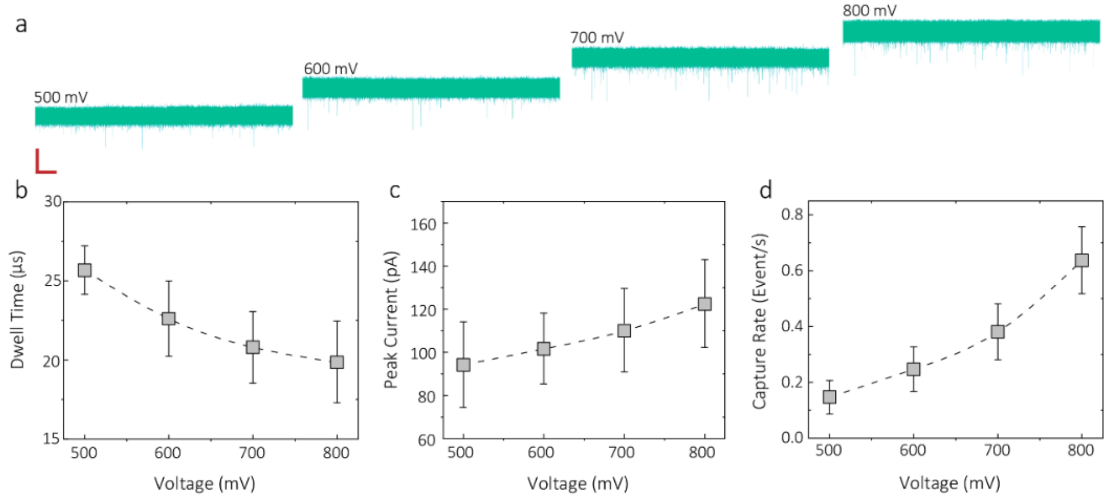

Figure S8. Statistics for the translocation of AuNP symmetrical dimers. (**a**) Current-time traces for the translocation of AuNP symmetrical dimer under applied voltages of 500, 600, 700, 800 mV. (**b**) Decrease of mean dwell times, (**c**) increase of mean peak currents, and (**d**) increase of event frequency are observed with increasing voltage. All translocation experiments were performed in 50 mM KCl 10 mM Tris-EDTA. Each current-time trace is recorded with a 1MHz sampling rate and filtered using a low-pass digital filter with a cut-off frequency of 100 kHz. Error bars represent the standard deviation of three independent experimental repeats with different nanopipettes. Scale bar: horizontal: 2 s, vertical: 100 pA.

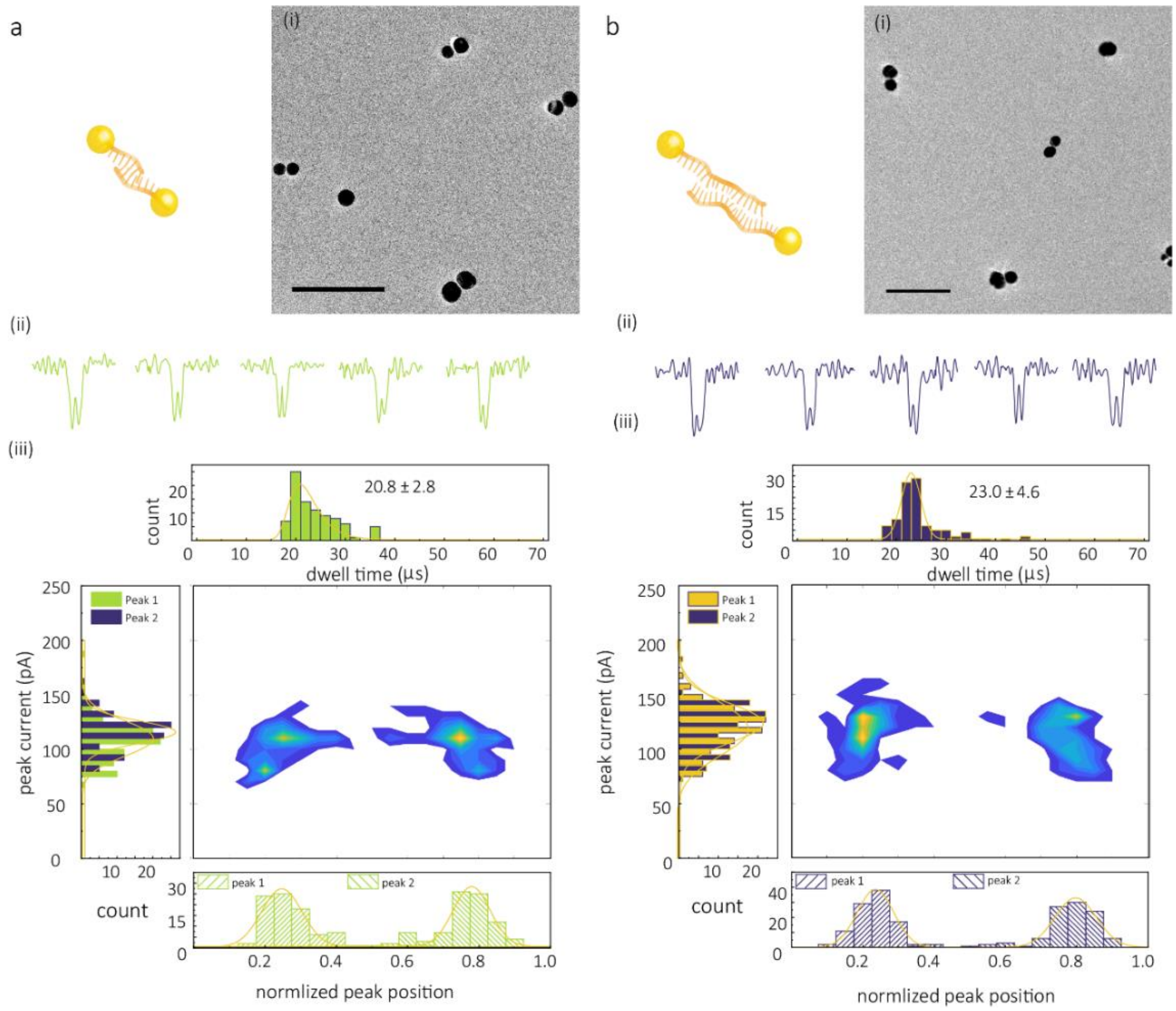

Figure S9. A comparison between translocation characteristics of AuNP symmetrical dimer with DNA bridge of different lengths. TEM images (Scale bar, 200 nm), individual events (Scale bar: vertical 50 pA, horizontal 20 μs), and statistics for (**a**) AuNP symmetrical dimer with 35 bases long DNA bridge (**b**) AuNP symmetrical dimer with 115 bases long DNA bridge. All translocation experiments were performed in 50 mM KCl 10 mM Tris-EDTA and at a voltage of -600 mV. The translocation data is recorded with a 1MHz sampling rate and filtered using a low-pass Bessel filter with a cut-off frequency of 100 kHz.

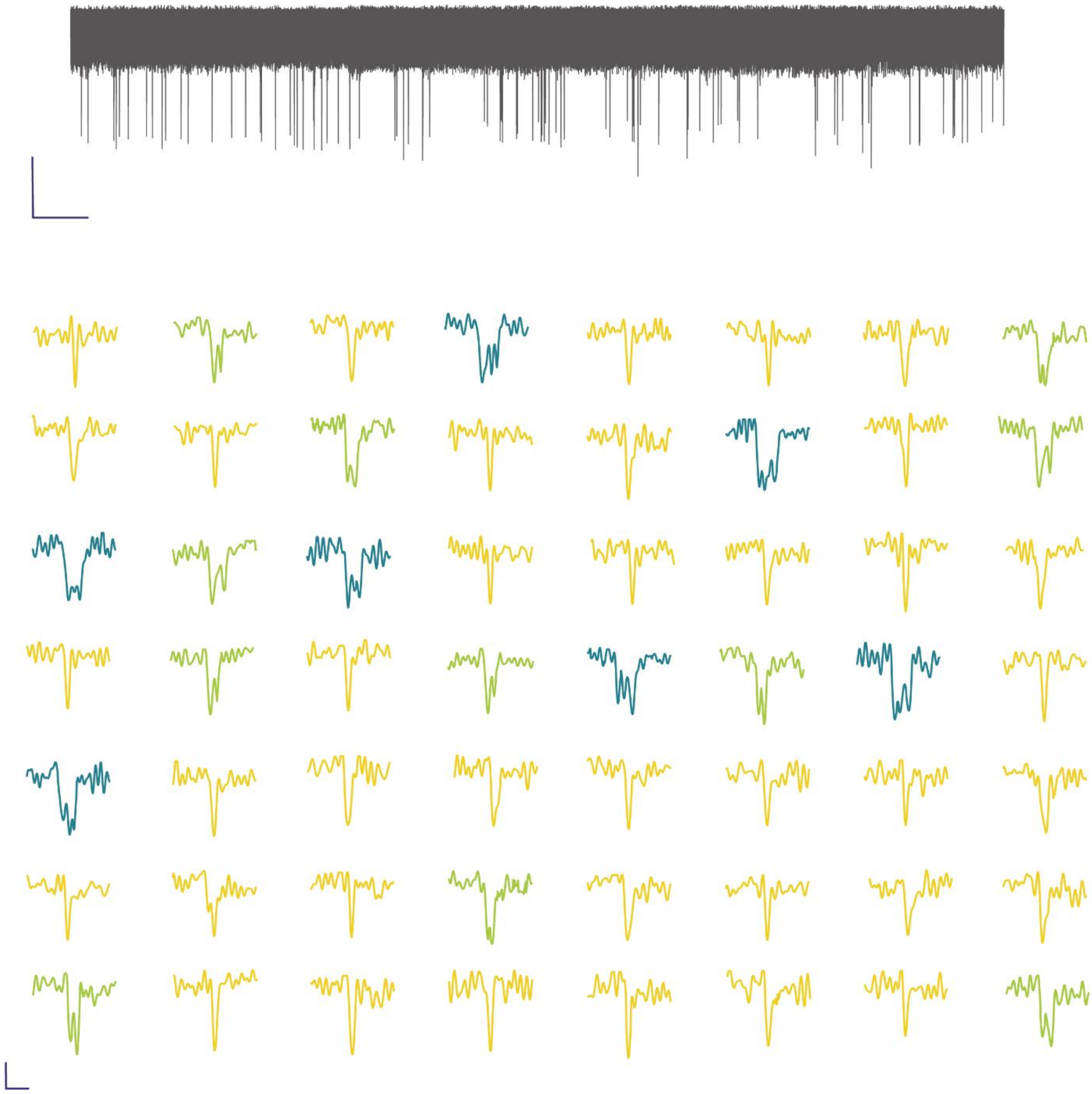

Figure S10. The current-time trace for a mixture for AuNP monomer, dimer, trimer. The first 56 events in this trace are listed below. Singlet, doublet, or triblets can be discriminated against. The scale bar for the current-time trace is 100 pA (vertical) and 10 s (horizontal); the scale bar for the individual events is 50 pA (vertical) and 20 μs (horizontal), respectively. The translocation experiments were performed in 50 mM KCl, 10 mM Tris-EDTA. The current-time trace is recorded with 1 MHz sampling rate and filtered using a low-pass Bessel filter with a cut-off frequency of 100 kHz.

Section S4. Single-particle SERS measurements

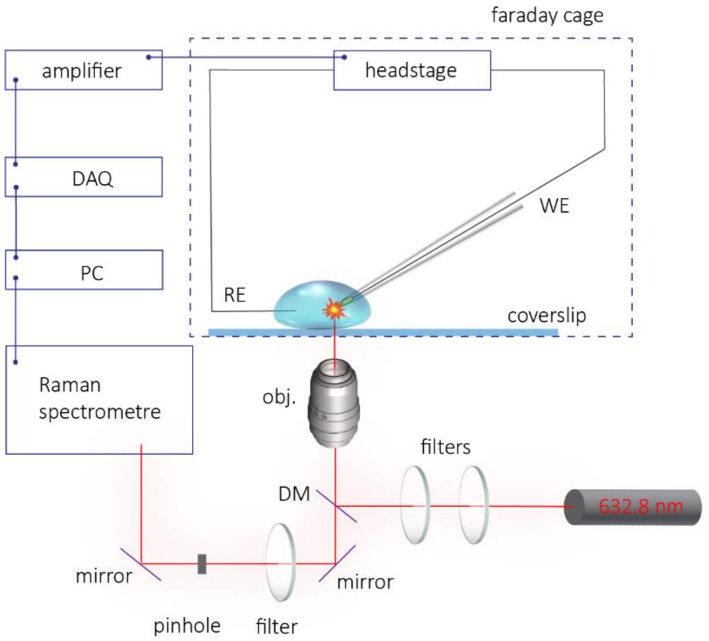

Figure S11. Customized electrical detection set-up with integrated laser optics to enable Raman spectroscopy. The Faraday cage containing a headstage of the amplifier, LED light, and WE/RE connectors. The cage had an opening at the bottom for the objective and was mounted on a motorized stage, adjustable in the x, y, and z directions. To minimize noise, the whole assembly was grounded and isolated from the stage. All experiments and recordings were conducted with the Faraday cage closed, and the lights switched off. The laser enters from the right-hand side of the inverted microscope and passes through two attenuation filters before entering a 40x air objective and illuminating the sample. The Raman spectra are collected by a spectrometer. (Shamrock SR-303i, Andor). A custom-made PTFE nanopipette holder is secured on a glass coverslip containing a droplet of KCl encased within a Kwik-cast gasket and hosts the Ground/RE. The nanopipette, filled with KCl and Au dimers, houses the patch electrode

Supplementary Note 3. Experiment details of single-particle SERS experiment

The fabrication of Au coated nanopipette

Single barrel quartz capillaries (o.d., 1.0 mm, i.d., 0.7 mm, Intracell) were plasma cleaned (Harrick Plasma) and pulled with a laser-based P-2000 pipette puller (Sutter Instruments) using a two-line program (heat 775, filament 4, velocity 30, delay 170, and pull 80; heat 825, filament 3, velocity 20, delay 145, and pull 130) to produce nanopipettes with nanopore diameters of approximately 45-55 nm as characterized by SEM imaging. It should be noted that the above pulling parameters are instrument-specific, and variations will exist from puller to puller. Then

the fabricated nanopipettes were held vertically by a customized metallic holder. A thin layer of Au (10 nm) was deposited on the cross-section of the nanopore using a Q150V Plus sputter coater (Quorum Technologies Ltd).

Single-particle SERS measurement

15 nM 4-ATP labeled dimers are loaded in the nanopipette (cis chamber) together with an Ag/AgCl patch electrode. An Ag/AgCl ground/reference electrode was placed in the bath located on a glass coverslip. The buffer used in the SERS measurements was 100 mM KCl 10 mM Tris-EDTA (pH=8). Current-time traces were recorded using an eOne XV amplifier (Elements) with a 100 kHz sampling rate and 10 kHz Bessel filter. When the particles pass through the laser-focused area, the Raman signal change can be recorded by a custom-built Raman microscope using a HeNe 632.8 nm excitation source, **Figure S11**.

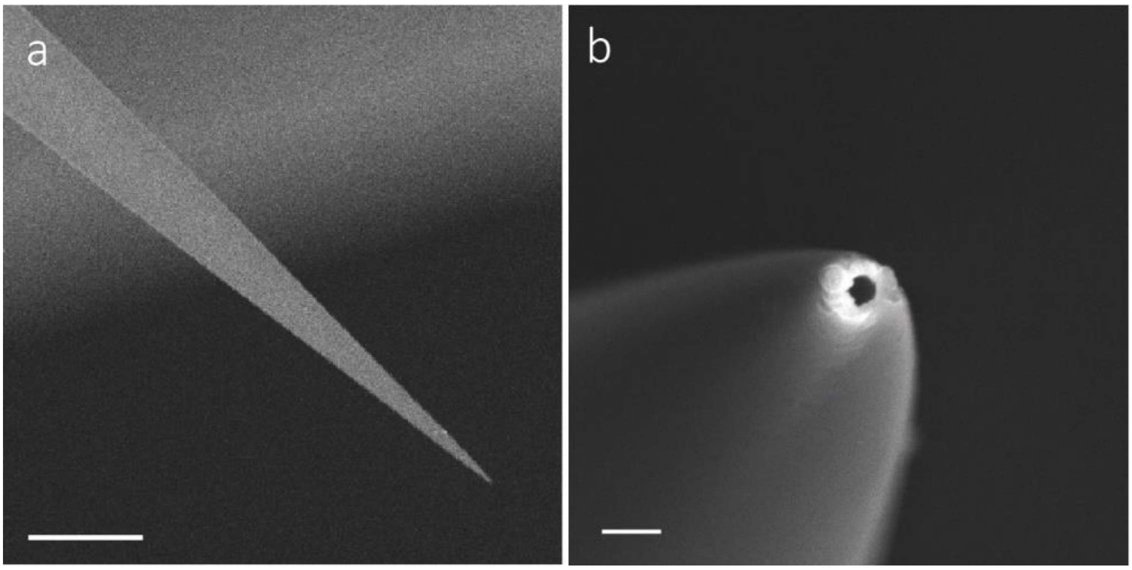

Figure S12. SEM images of Au coated nanopipette (**a**) side view (scale bar, 10 µm), (**b**) top-view (scale bar, 200 nm). The size of the nanopore at the tip was about 50 nm.

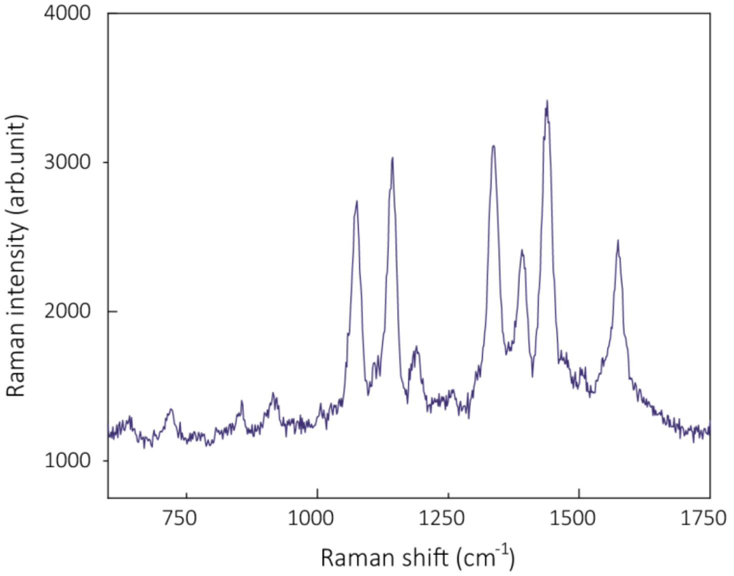

Figure S13. SERS spectrum of 4-ATP present within the nanogap of AuNP symmetrical dimers. The spectrum of 4-ATP shows expected vibrational bands at 1138, 1387, and 1571 cm^{-1}.

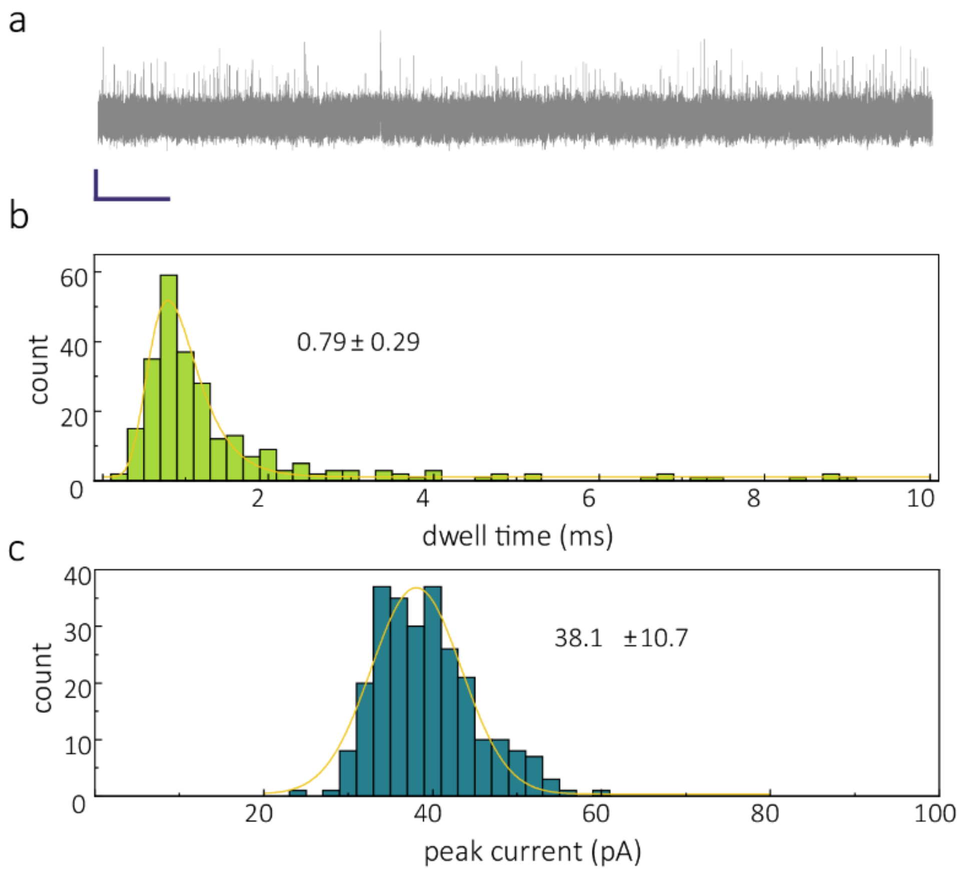

Figure S14. Translocation of PEG stabilized 35 nm AuNP dimers functionalized with 4-ATP. The current-time trace (Scale bar: vertical 50 pA, horizontal 5 s, and statistics for 1 nM Raman sample (35 nm AuNP Dimer functionalized with 4-ATP dye, protected by SH-PEG). All the translocation experiments were performed in 100 mM KCl, 10 mM Tris-EDTA. The current-time trace is recorded with a 100 kHz sampling rate and filtered using a low-pass Bessel filter with a cut-off frequency of 10 kHz. The mean dwell time (0.79 ± 0.29 ms) was longer than for non-PEG modified dimers due to molecules has a higher viscosity and lower charge.

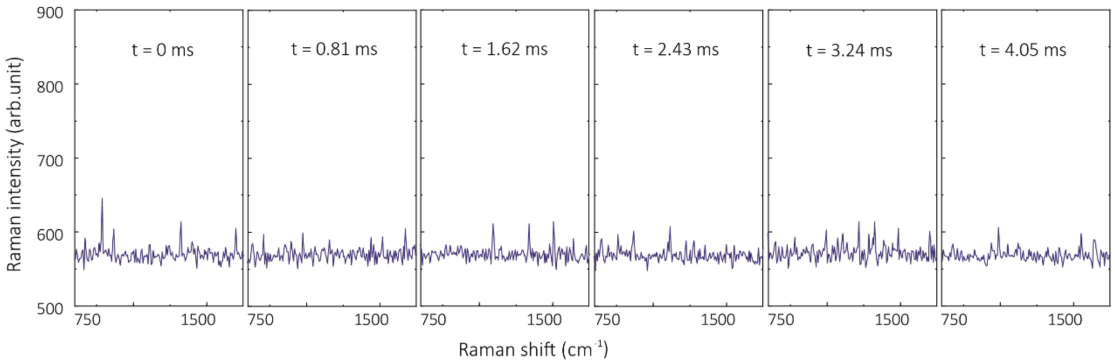

Figure S15. Control experiment for single-molecule SERS detection. An example of Raman spectra of PEGs modified 4-ATP loaded Au dimer using +800 mV voltage. There is a minimal enhancement (Raman intensity around 50) in vibrational bands attributed to the momentarily interaction of the AuNP dimer with the nanopore, but no translocation occurs.

Section S5. Molecular probes for single-molecule detection of miR-141-3p

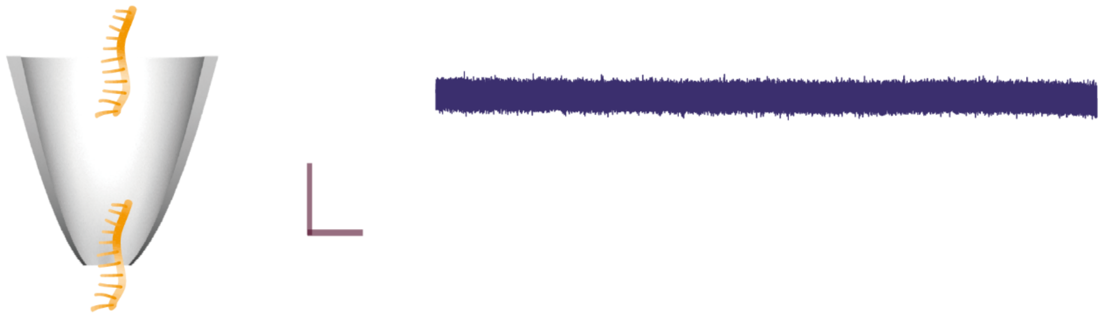

Figure S16. Sensing of miRNA without AuNP probes. The current-time trace of miR-141-3p (10 nM) nanopore sensing (Scale bar: vertical 50 pA, horizontal 5 s). The translocation experiments were performed in 50 mM KCl,10 mM Tris-EDTA, and at a voltage of -600 mV. The current-time trace is recorded at a 1MHz sampling rate and filtered using a low-pass Bessel filter with a cut-off frequency of 100 kHz. There are no observable translocations.

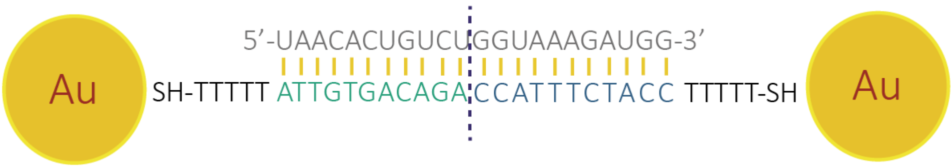

Figure S17. Schematic of miR-141-3p and AuNP monomer probes. The miR-141-3p detection probes contain two types of ssDNA functionalized AuNP monomers. Each of them was modified by an 11 base recognition chain, which can hybridize with half of 22-base-long miR-141-3p (grey sequence in the figure). With the addition of the target, the monomer probes form a dimer. It is noteworthy that there are 5T close to the ssDNA recognition sequence to minimize the steric hindrance.

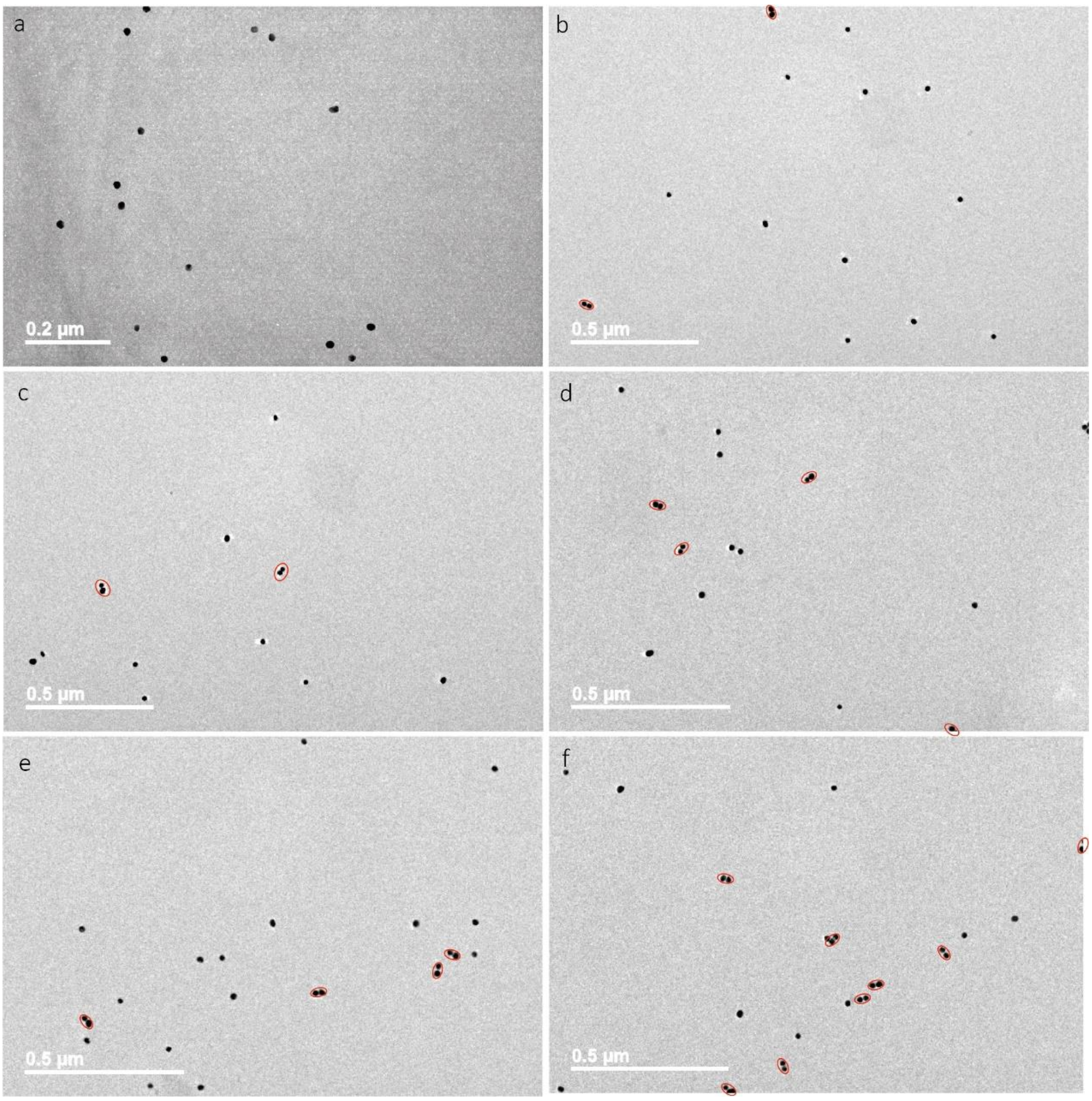

Figure S18. TEM images of AuNP monomer probes incubated with different levels of miRNA. The TEM images (scale bars, 200 nm) of AuNP monomer probes incubated with (**a**) 0 nM (**b**) 10 pM (**c**) 100 pM (**d**) 1 nM (**e**) 10 nM (**f**) 100 nM miR-141-3p.

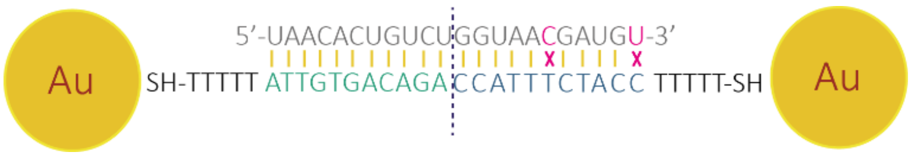

Figure S19. The schematic of a miR-141-3p probe binding to miR-200a-3p. This was used as a control experiment and consisted of a two-base mismatch, which heavily decreases the affinity of hybridization.

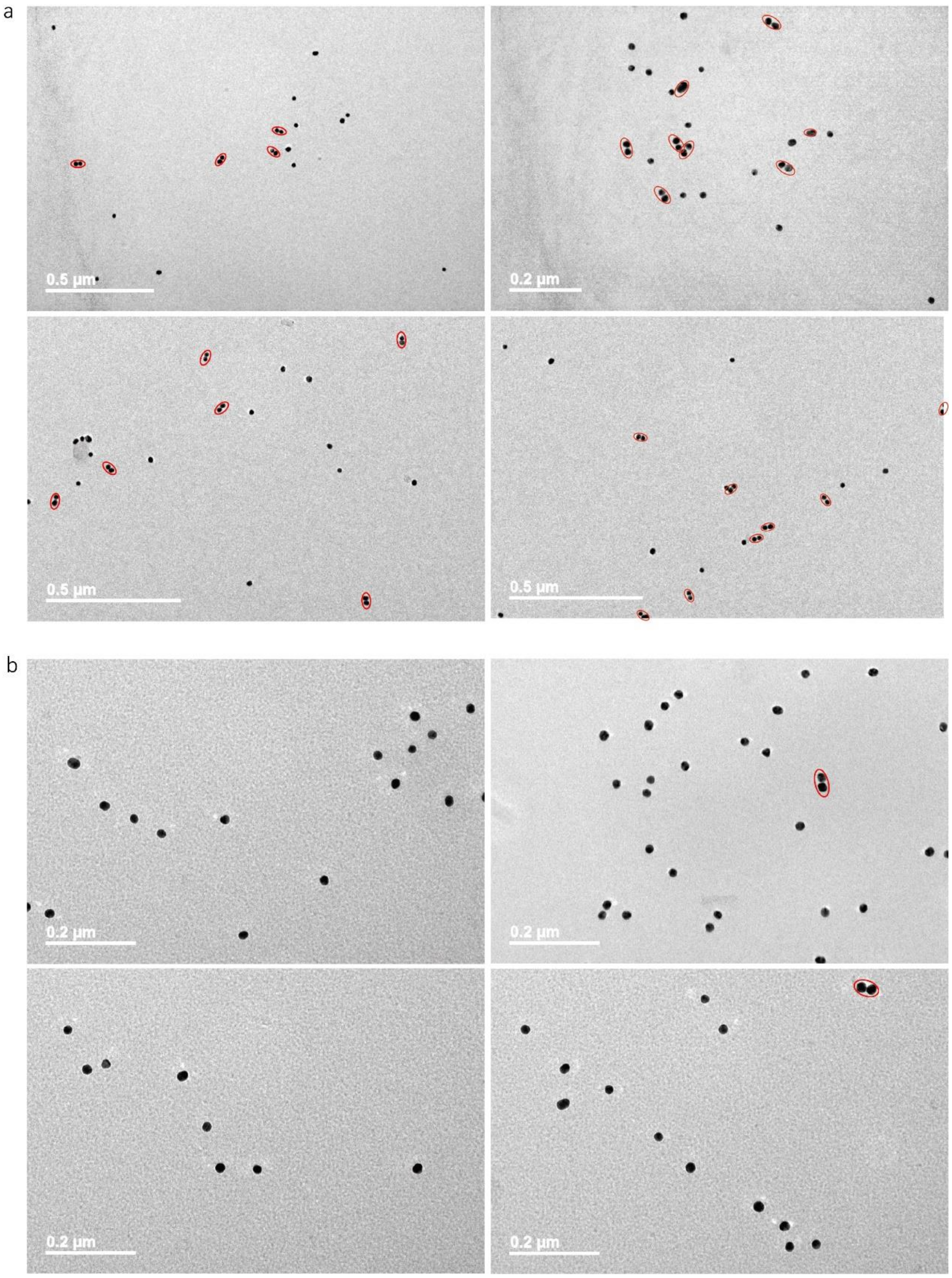

Figure S20. TEM images of the AuNP monomer miR-141-3p probe with the addition of miR-141-3p and miR-200a-3p. A large number of miR-141-3p probes (2 nM) dimerized to dumbbell molecules with the presence of (**a**) 100 nM miR-141-3p, which is fully matched with the probe sequence, where only very few dimer molecules generated with the addition of (**b**) 100 nM miR-200a-3p, which has two base mismatch with the AuNP probe.

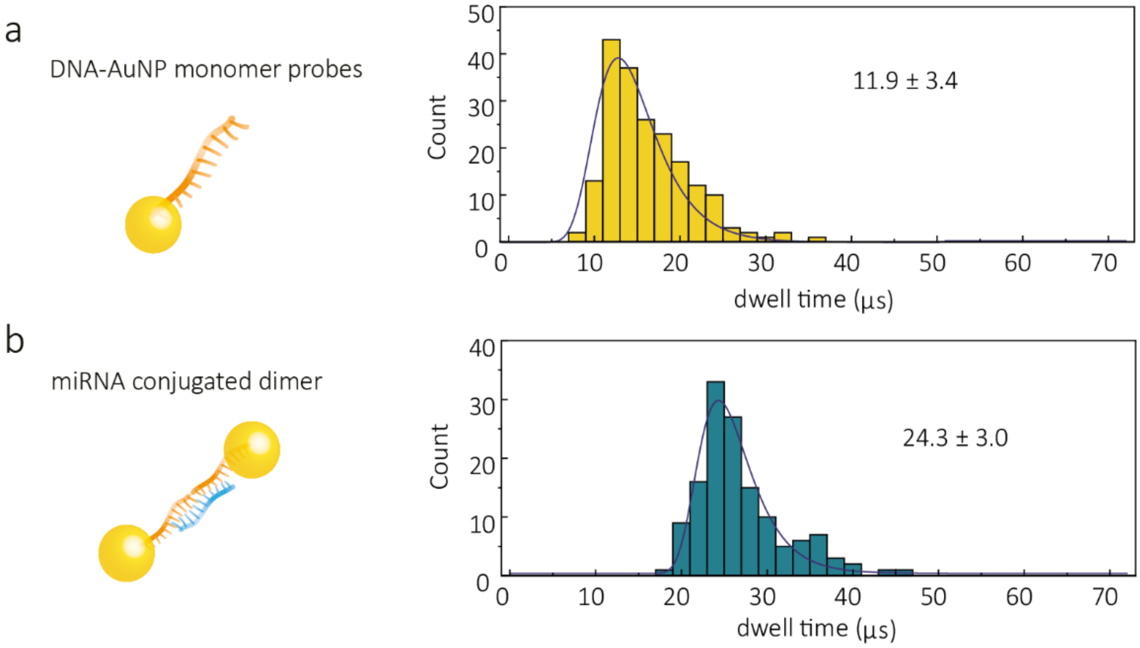

Figure S21. Dwell times for the translocation of (a) DNA-AuNP monomer probes (b) miRNA conjugated dimers.

Section S6. Molecular probes for single-molecule detection of PCT

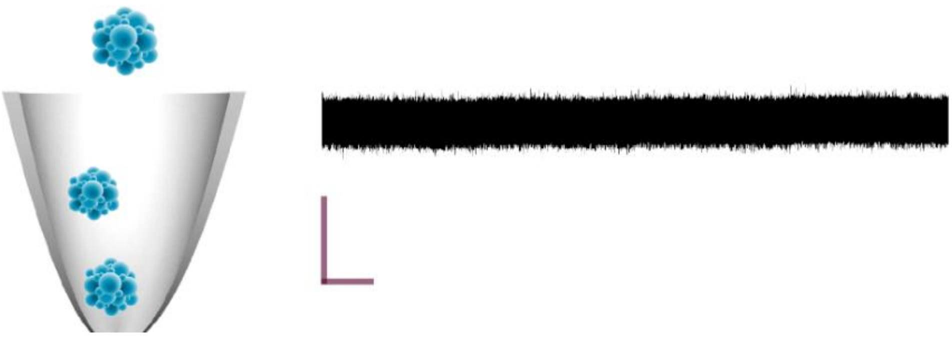

Figure S22. Current-time trace for detection of PCT (100 nM) (Scale bar: vertical 50 pA, horizontal 5 s). The translocation experiments were performed in 50 mM KCl,10 mM Tris-EDTA, and at a voltage of -600 mV. The current-time trace is recorded at a 1MHz sampling rate and filtered using a low-pass Bessel filter with a cut-off frequency of 100 kHz. Even with this high concentration of PCT, no translocation signal can be distinguished due to the low signal-to-noise ratio.

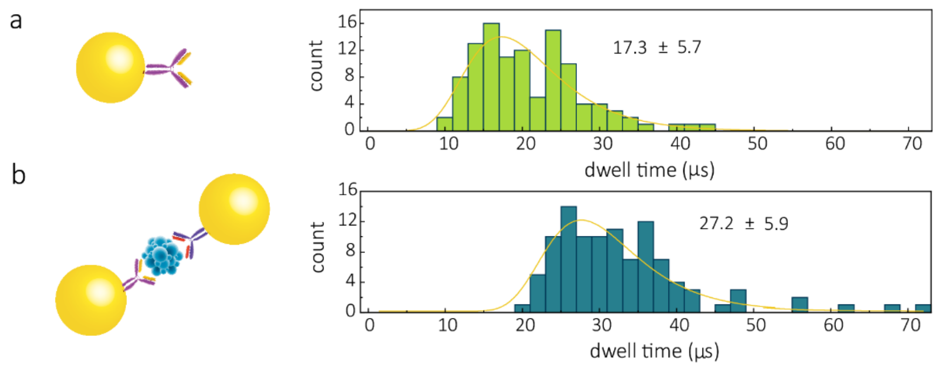

Figure S23. Histogram of the translocation dwell times of AuNP monomer PCT probes in or without the presence of PCT. The dwell time of the translocation of (**a**) AuNP monomer PCT probes (**b**) The conjugated dimer was linked by an antibody-antigen-antibody sandwich bridge.

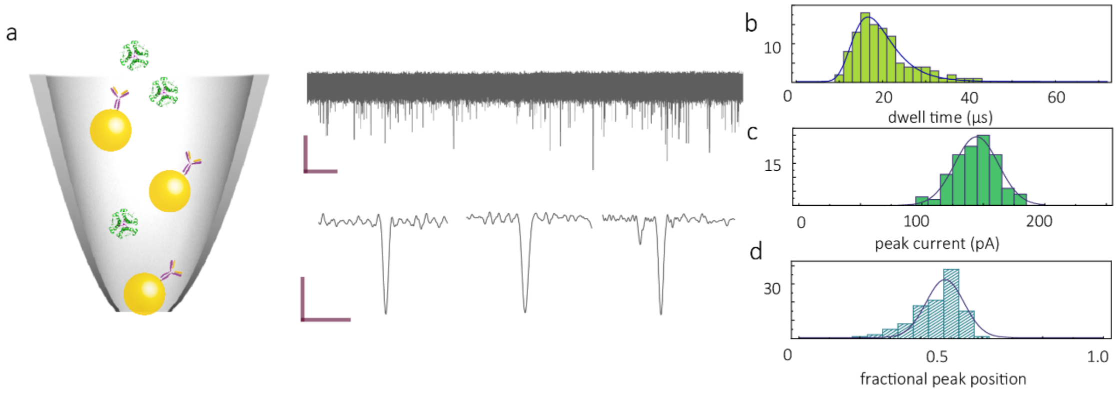

Figure S24. Control experiments for the AuNP monomer PCT probe. The current-time trace (Scale bar: vertical 50 pA, horizontal 5 s), individual events (Scale bar: vertical 50 pA, horizontal 20 μs), and statistics for 1 nM AuNP monomer PCT probes incubated with 1 nM insulin. All the translocation experiments were performed in 50 mM KCl 10 mM Tris-EDTA. The current-time trace is recorded at a 1MHz sampling rate and filtered using a low-pass Bessel filter with a cut-off frequency of 100 kHz. There are no double peak events but single peak events corresponding to free PCT probes.

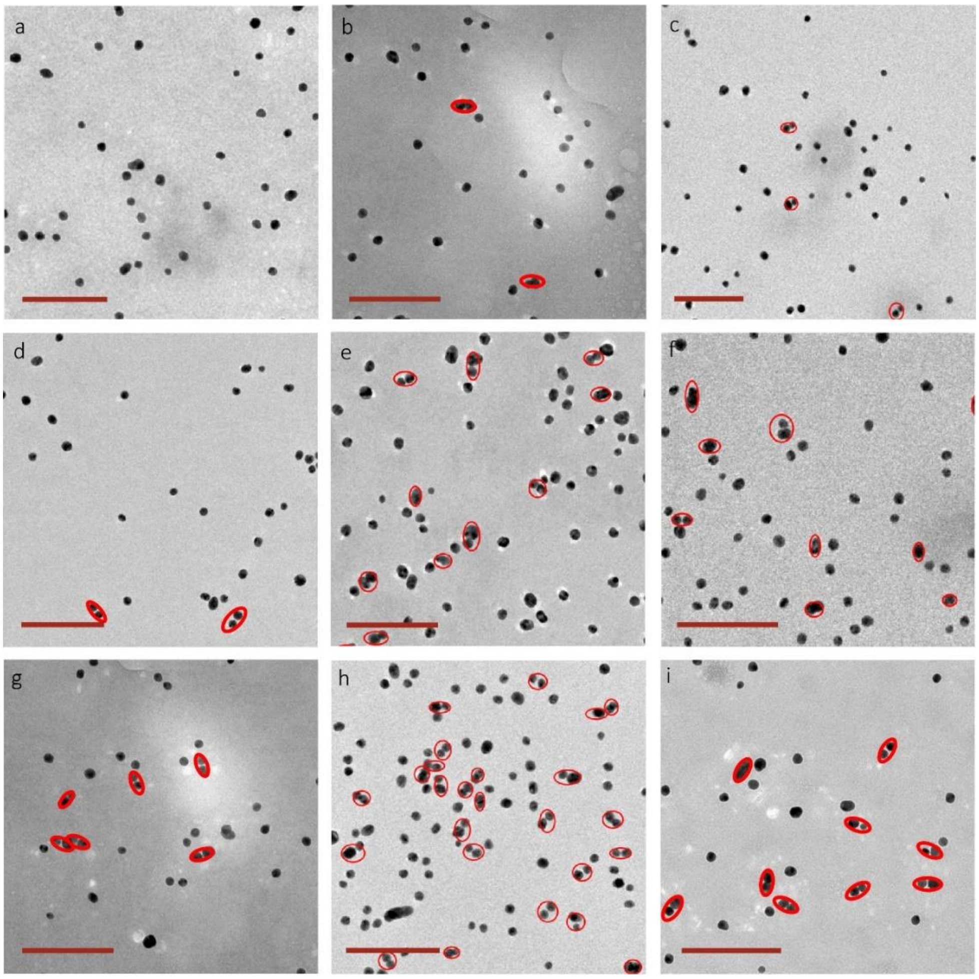

Figure S25. TEM images of AuNP monomer probes incubating with different levels of PCT. The TEM images (scale bars, 200 nm) of 2 nM AuNP monomer probes (1 nM for each type) incubated (**a**) without PCT and with (**b**) 40 pM, (**c**) 80 pM, (**d**) 400 pM, (**e**) 4 nM, (**f**) 20 nM, (**g**) 40 nM, (**h**) 100 nM, and (**i**) 200 nM PCT.

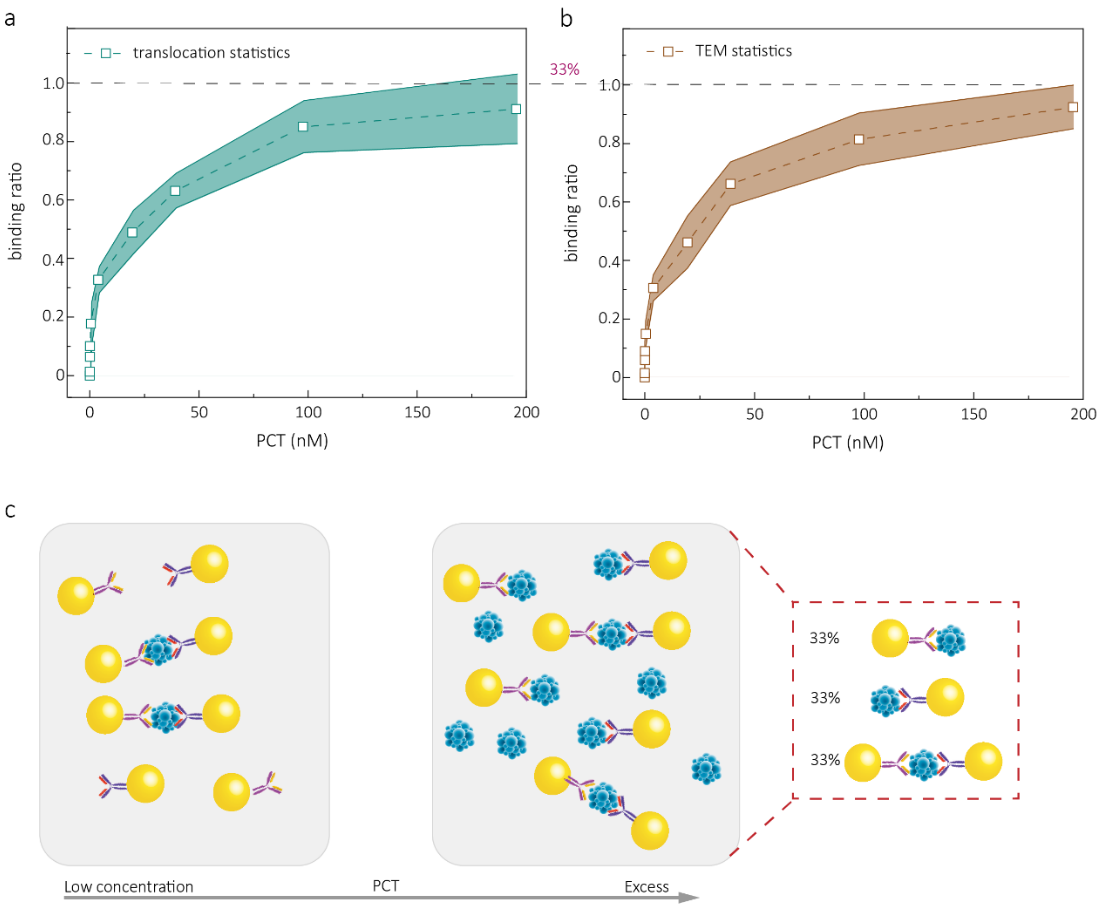

Figure S26. PCT binding curve obtained by translocation and TEM nanoparticle counting. The binding assay of 1 nM PCT monomer probes with PCT ranging from 0 to 200 nM. (**a**) The binding ratio is calculated from the percentage of double peak translocation signatures in all signals. Error bars represent the standard deviation of three independent experimental repeats with different nanopipettes. (**b**) binding ratio vs. PCT concentration as calculated by TEM counting of dimers. Error bars represent the standard deviation of three independent experimental repeats. The binding curve has been normalized to the maximum probable binding ratio. (**c**) Schematic of various variations of the NPs and antigen in solution. The probability of detecting a dimer is 33%. Aside from the TEM and nanopore data shown above, this value was also verified using a one-step sandwich immunoassay. All data in the manuscript was normalized to 1 based on the maximum probable value.

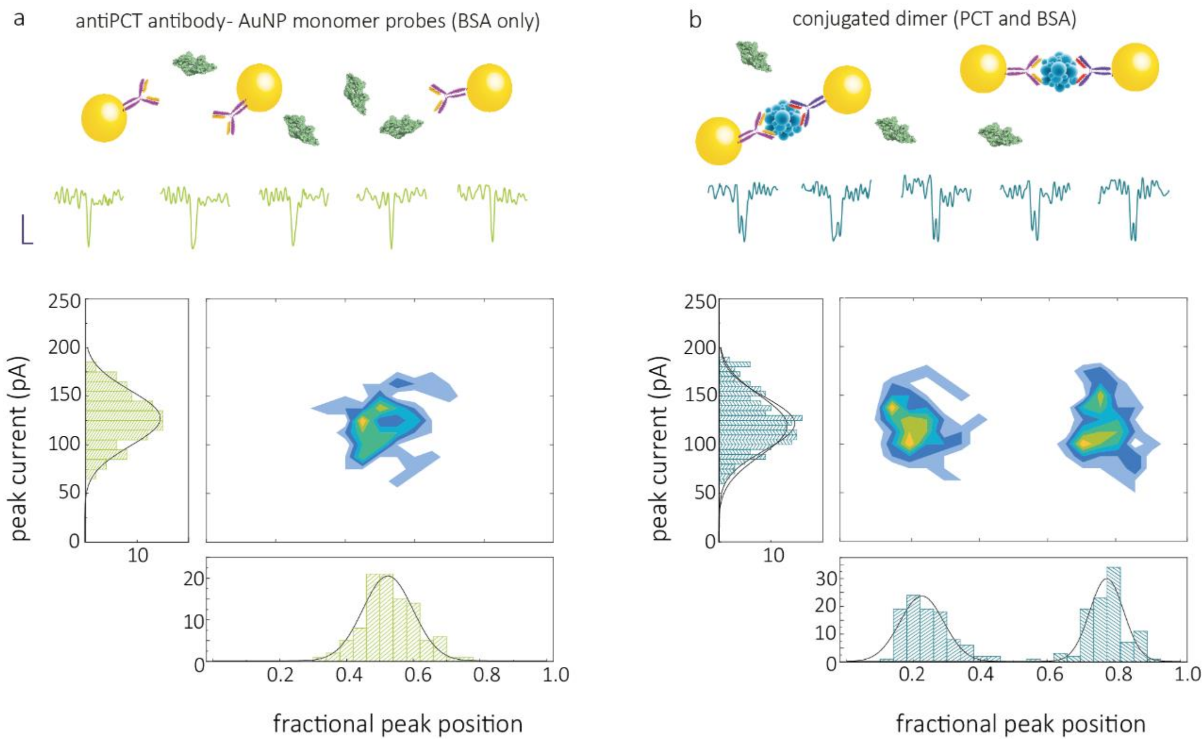

Figure S27. Translocations in a background medium consisting of a high concentration of BSA. (a) 2 nM antiPCT antibody-AuNP translocation events and statistic results in a 1% BSA solution. The result shows that the particle is stable in the highly concentrated BSA solution. (b) The PCT binding experiment in 1% solution. In the presence of PCT (10 ng/ml), the conjugated molecules pass through the nanopore and show double peak events. Scale bar: horizontal: 10 µs; vertical: 100 pA. All translocation experiments were performed in 50 mM KCl 10 mM Tris-EDTA and at a voltage of -600 mV. The translocation data is recorded with a 1MHz sampling rate and filtered using a low-pass Bessel filter with a cut-off frequency of 100 kHz.

Section S7. Data analysis

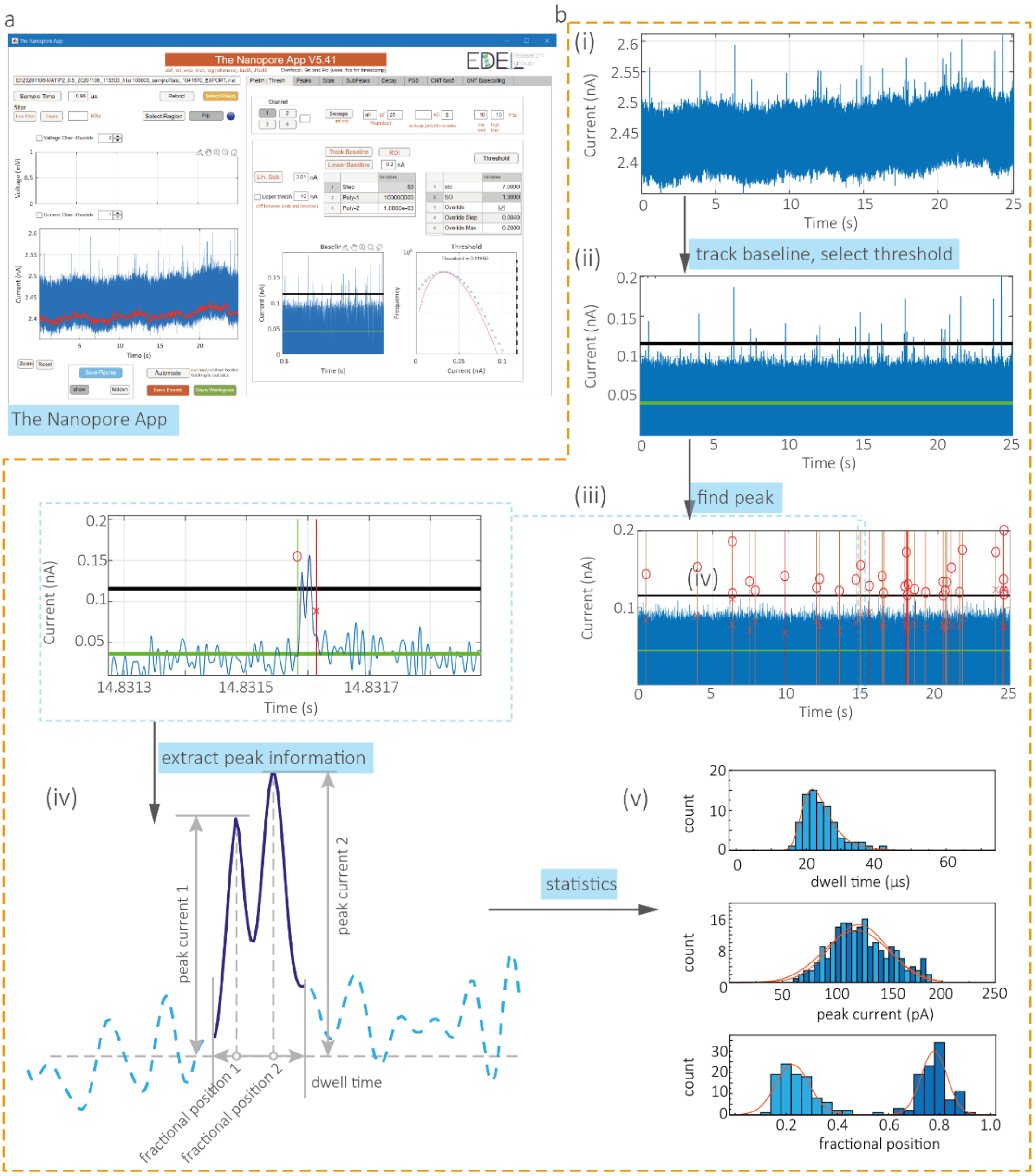

Figure S28. The flow chart for the data analysis.

Data analysis was performed as follows:

1. Current-time trace loaded into custom written matlab script, **Figure S28 b(i)**.

2. Digital refiltering and resampling. The events were recorded unfiltered at the maximum sampling rate. Using the app, the trace can be refiltered or resampled to better visualize the events and improve the signal-to-noise ratio. In this paper, most data were refiltered using a 100 kHz low-pass filter and resampled at 1 MHz.

3. Baseline tracking, **Figure S28 b(ii)**. The baseline was tracked to compensate for fluctuations in the signal enabling improved classification of events.
4. Event classification using a threshold. An all points histogram was used to fit a Poisson distribution to the noise. To classify events, a threshold of at least 6σ was used.
5. Peak selection, **Figure S28 b(iii)**. Events with peak currents above the threshold were selected. Several conditions can be imposed to help with selection. For example, normally, the event time is defined by using a full width at half maximum (FWHM) of the translocation signal.
6. Single molecule statistics **Figure S28 b(iv)**. After peak selection, the whole peak information including dwell time, area, deltaT (the time interval between two successive events) and the subpeak information (peak height, normalized fractional position) was obtained.
7. Exporting statistical results, **Figure S28 b(v)**. In this paper, dwell time, peak current, fractional peak position was exported.

Supplementary Reference

[1] a) L. M. Demers, C. A. Mirkin, R. C. Mucic, R. A. Reynolds, R. L. Letsinger, R. Elghanian, G. Viswanadham, *Anal. Chem.* **2000**, 72, 5535; b) S. J. Hurst, A. K. R. Lytton-Jean, C. A. Mirkin, *Anal. Chem.* 2**006**, 78, 8313.

[2] S. Lee, Y. H. Zhang, H. S. White, C. C. Harrell, C. R. Martin, *Anal. Chem.* **2004**, 76, 6108.

[3] a) Y. X. Wang, K. Kececi, M. V. Mirkin, V. Mani, N. Sardesai, J. F. Rusling, *Chem. Sci.* **2013**, 4, 655; b) E. A. Heins, Z. S. Siwy, L. A. Baker, C. R. Martin, *Nano Lett.* **2005**, 5, 1824.

[4] J. Y. Y. Sze, A. P. Ivanov, A. E. G. Cass, J. B. Edel, *Nat. Commun.* **2017**, 8.
